# IC-Behavior: An interdisciplinary taxonomy of behaviors

**DOI:** 10.1371/journal.pone.0252003

**Published:** 2021-09-17

**Authors:** Kai R. Larsen, Lauren J. Ramsay, Cristina A. Godinho, Victoria Gershuny, Dirk S. Hovorka

**Affiliations:** 1 University of Colorado, Boulder, Colorado, United States of America; 2 University of Colorado, Denver, Colorado, United States of America; 3 Católica Research Centre for Psychological—Family and Social Wellbeing, Universidade Católica Portuguesa, Lisbon, Portugal; 4 University of Arizona, Tucson, Arizona; 5 University of Sydney, Sydney, Australia; Universitat Luzern, SWITZERLAND

## Abstract

Academic disciplines are often organized according to the behaviors they examine. While most research on a behavior tends to exist within one discipline, some behaviors are examined by multiple disciplines. Better understanding of behaviors and their relationships should enable knowledge transfer across disciplines and theories, thereby dramatically improving the behavioral knowledge base. We propose a taxonomy built on the World Health Organization’s *International Classification of Functioning*, *Disability*, *and Health* (ICF), but design the taxonomy as a stand-alone extension rather than an improvement to ICF. Behaviors considered important enough to serve as the dependent variable in articles accepted for publication in top journals were extracted from nine different behavioral and social disciplines. A six-step development and validation process was employed, leading to the final taxonomy. A hierarchy of behaviors under the top banner of *Engaging in activities/participating*, reflective of ICF’s D. hierarchy was constructed with eight immediate domains addressing behaviors ranging from learning, exercising, self-care, and substance use. The resulting *International Classification of Behaviors* (*IC-Behavior*), provides a behavior taxonomy targeted towards the interdisciplinary integration of nomological networks relevant to behavioral theories. While *IC-Behavior* has been labeled v.1.0 to communicate that it is by no means an endpoint, it has empirically shown to provide flexibility for the addition of new behaviors and is tested in the health domain.

## Introduction

The behavioral sciences are often organized around the behaviors they address. While providing a powerful lense for disciplinary focus, such arrangements reduce the potential for theory integration and extension. In this article, we design an interdisciplinary classification of behaviors, but focus on its applicability to the health domain. A large number of human behaviors and actions have health implications. Health-promoting behaviors are associated with decreased mortality and morbidity [1]. Health-impeding behaviors, on the other hand, are associated with increased mortality and morbidity. While certain health behaviors, such as smoking, are relatively well understood within their contexts [2] and this knowledge has led to progress in changing these behaviors [e.g., 3], whole classes of behaviors are often excluded by behavioral health researchers. For instance, anti-social and criminal behaviors are seldom considered in the construction of behavioral medicine frameworks, although they have severe health implications for victims, perpetrators, their respective families, and the public at large.

While progress has been made in understanding and evaluating health behaviors [e.g., 4–8], research has typically focused on a small set of behaviors. Behavior taxonomies often focus on evaluating which behaviors have the greatest health implications; such representations, in turn, are further limited in that they are usually found by and generated for behavioral medicine researchers and practitioners [e.g., 5]. Suggesting an interdisciplinary path, Kasl and Cobb [9] found that “sick role behavior, then, appears to have some significant nonmedical determinants.” Moreover, Nudelman et al. [4] stated, “Notwithstanding the differences between [health behaviors], considering them as completely distinct from each other is also incompatible with evidence.” An exclusive focus on health behaviors may deprive researchers of the connections among all human behaviors, thus limiting their understanding of how behaviors interact and change. We therefore suggest that behaviors exist as part of a complex system [10], and that interdisciplinary behavior classifications or taxonomies of general behavior frameworks are needed to move research forward.

## Research motivation

While we retain the term ‘classification’ from ICF, we will refer to the end-product of our work as a taxonomy. A taxonomy is a statement of the entities that exist in a domain and their hierarchical relationships to each other; it demonstrates a commitment to using a shared language when referring to the entities in the domain [11,12]. Using this definition, we submit that creating a broad interdisciplinary taxonomy of behaviors will (1) increase understanding and refine descriptions of individual behaviors, (2) heighten awareness of hierarchies and networks of behaviors, (3) enable behavior change and longitudinal tracking, and (4) facilitate interdisciplinary knowledge-transfer.

First, it is important to understand the characteristics of behaviors [5]. It has been established that multiple categories of co-occurring health behaviors exist, but “there presently is no consensus regarding the number or precise content of the categories required to describe these behaviors” [13]. As Michie et al. [14] suggested, unless there is precision in describing and defining the behavior that needs to be changed, it will not be possible to identify construct domains that might explain the underlying behavioral processes and the associated opportunities for behavior alteration. There is therefore a need for careful definition and specification of behaviors within behavioral and social science studies.

With regard to longitudinally changing behaviors, McEachan et al. [5] indicated the importance of classifying behaviors as a way to learn how to effectively intervene in order to change different *groups* of behaviors, rather than targeting a single behavior [15]. Focusing on hierarchies and networks of behaviors could drive new scientific understandings of behavior “cascades,” where the focus would be on identifying small behavioral changes that have large impacts on health-impeding behaviors. For example, to help an individual quit smoking, a focus on other behaviors, such as exercise, might lead that person to naturally understand that smoking inhibits physical performance as they moved from no exercise to walking and then to running [16]. Continued exercise, in turn, may reveal to the same individual that other behaviors (e.g., eating junk food) are detrimental to their running progress [17]. Therefore, helping an individual to begin exercising may trigger a whole behavior cascade that changes the person’s life. It is not clear that helping someone to stop smoking would in itself encourage them to start exercising; in fact, people who quit often experience weight gain [18], which can decrease their motivation to continue cessation behavior [19–21].

A less frequently suggested benefit of developing a comprehensive behavior taxonomy is the potential for transfer of knowledge bases across disciplines, including theories of behavior and behavior change approaches [5]. If we knew how to transfer knowledge about behaviors across theories—or theoretical knowledge across behavior modification approaches—all behavioral disciplines would advance more quickly.

## Starting point: The ICF

The World Health Organization’s (WHO) *International Classification of Functioning*, *Disability*, *and Health* [ICF, 22] is a framework describing health and health-related states that has been cited over 10,000 times. It consists of a classification system that can be used for multiple purposes across different sectors, and functions as a standardized tool for research in all aspects of day-to-day human functioning.

In the ICF, disability and functioning are conceptualized as the product of interactions between health conditions (e.g. diseases, disorders and injuries) and contextual factors. It is comprised of four higher-level categories (see https://apps.who.int/classifications/icfbrowser/): (1) *b—Body functions* (i.e., physiological functions of body systems, including psychological functions); (2) *s—Body structures* (i.e., anatomical parts of the body such as organs, limbs and their components); (3) *d—Activities and participation* (i.e., execution of a task or action by an individual or their involvement in a life situation); and (4) *e—Environmental factors* (the physical, social and attitudinal environment in which people live and conduct their lives); and (These categories allow one to describe disability in terms of changes in body function and structure (the b—and s—domains), as well as in terms of functioning levels relative to what people with specific health conditions can do in a standard environment and what they actually do in their usual environment (within the Activities and participation domain, d -). The environmental factors that interact with all these components are also included as a domain (e -). These higher-order categories are subdivided in up to three lower-order levels, forming a complex and hierarchical tree-like structure.

In 2001, the ICF was endorsed officially by all 191 WHO member states as the international standard for description and classification of health and disability. In addition, it exists on the BioPortal, a comprehensive repository of biomedical ontologies. The ICF is thus a flexible and comprehensive framework that offers a systematic classification scheme. Hence, the ICF, specifically its *D*. *Activities and Participation* hierarchy, was selected as the best starting point for a behavior taxonomy. That said, existing literature on the ICF suggests that there is potential for improvement [27] and further development so that the framework encompasses behaviors related to non-health-related domains.

### Aim

The aim of the present study was to draw upon the ICF to create a generalizable and extensible interdisciplinary taxonomy of behaviors, in order to improve our understanding of the interconnected nature of behaviors.

## Methods

The *IC-Behavior* taxonomy-building process consisted of several separate rounds of analysis and category-construction exercises in six different steps: (1) extraction of behaviors (variables) from papers; (2) initial categorization of behaviors; (3) ICF appropriation (top-down categorization); (4) hierarchy testing and refinement; (5) taxonomy evaluation; and (6) evaluation of the current taxonomy against past behavioral medicine taxonomies.

For this process, the following principles of taxonomy development were employed:

1. We extended from a solid starting point by building our taxonomy on the existing international agreement embedded in the ICF;
2. We used vetted and defined behaviors; the data used to extend the taxonomy were published as part of behavioral work in top journals across nine disciplines; and
3. We carefully developed terminology, linking each behavior to existing ontologies (e.g., the NCI Thesaurus and the NCI Metathesaurus).

Each of the six rounds, which are described below, were conducted with different experts.

### Step 1: Extraction of behavior variables from papers

Behavior representations (variables and their definitions) in nine disciplines were extracted from top journals determined by journal rankings, citation factors, and interviews with faculty in each discipline. These were collected over a 10-year period by a National Science Foundation-funded project named the Human Behavior Project (HBP; see http://www.theorizeit.org for details). HBP did not aim to create a generalizable set of articles; instead, it collected items containing behavioral constructs from the top journals in each of the nine disciplines. This selection procedure is similar to the approach used by databases such as the Social Science Citation Index, in that the database is only comprised of journals that meet a certain level of quality. It should be noted that while this approach leads to lower generalizability, it presumably reflects a higher quality of research and behavior specification; however, we acknowledge that there may be bias built into the rating processes used to rank journals. S1 File contains a list of journals, years covered, and disciplines.

Behaviors were defined as “anything a person does in response to internal or external events. Actions may be overt (motor or verbal) and directly measurable, or covert (activities not viewable but involving voluntary muscles) and indirectly measurable; behaviours are physical events that occur in the body and are controlled by the brain” [7]. Differences in frequencies and intensities of behaviors were ignored. Every article in an included year was examined using the following inclusion criteria:

1. Articles had to test quantitative models at the individual level of analysis. All behaviors had to be at the individual level; thus, the actions of organizations or groups of people were not included. An example of such a group variable was *adoption of the principles of effectiveness* [23], defined as “Whether a school district adopted the Principles of Effectiveness program by conducting an evaluation of their program.” This kind of behavior occurs at an organizational level and subsumes too many individual-level behaviors to be classified accurately.
2. Articles had to contain at least one construct as well as at least one behavior.
3. The behavior had to be a dependent variable in a quantitative model and of practical interest.
4. The behavior had to be observable.

The exclusion criteria for the current study were as follows:

1. Variables that measure outcomes of behaviors were not included because of the focal person’s lack of control over them. Such variables included consequences of behaviors, such as arrests based on the behavior of committing a crime, or developing an ability to do something from practicing the skill. There are a multitude of factors that contribute to such outcomes, some of which are beyond the participants’ control, which runs counter to the definition of “behavior” employed in this study.
2. Behaviors that are proxies for other variables were not included due to their lack of relevance outside of an experimental setting. An example of such a variable is *extra role service behavior* [24], defined as “Whether the participant answered the phone after the simulation was complete.” These variables were excluded, because including them in the classification process would decrease the external validity of the hierarchy and its use in a real-world behavioral intervention setting.
3. Autonomic behaviors (e.g., breathing) were excluded.
4. Behaviors that required a rating beyond whether they occurred were excluded.

The data collection team consisted of carefully trained undergraduate and master’s level graduate students at a large, western U.S. research university. In all, 5,461 articles containing at least one behavioral construct were retained for further examination. For the IC-Behavior taxonomy, we focused on dependent variables (the end-point in behavioral theories or analyses) because these are often behaviors considered worthy of specific attention, and many disciplines define themselves in terms of the dependent variables they examine. A total of 1,626 dependent variables were found that were classified as behaviors and a random set of 813 variables were examined. Many variables were split because they contained groups of behaviors (such as *exercise* followed by a list of examples like *running*, *swimming*, *yoga*, etc.), resulting in a set of 1,180 individual behaviors. The definitions for all variables were carefully reexamined in the original paper and updated as needed. The remaining set of behaviors was retained for future expansion and validation purposes.

### Step 2: Initial categorization of behaviors

To become familiar with the behaviors and their definitions, their synonymous terms, and their relationships, our research team conducted a bottom-up categorization exercise. Our goals here were to gain a better understanding of the behaviors and reduce the set of 1,180 behaviors by removing duplicates. We here define *categorization* as corresponding to “a cognitive activity that leads the individual to treat different objects in the same way, in order to move beyond specificities toward generality” [25].

For this exercise, we employed a team of six experienced research assistants (operating in pairs) and a faculty member (working independently). Each categorization decision was proposed by one team, confirmed or changed by another team, and—if confirmed—examined by the faculty member. The process took approximately 40 hours. Identical variables were combined, and variables referring to the same general type of behavior, sometimes at different levels of abstraction, were merged into small hierarchies.

Once this initial step was finished, the WHO’s *International Classification of Functioning Disability and Health* was used to further classify these behaviors. The small hierarchies were used to expand ICF categories as needed.

### Step 3: ICF appropriation

The *Oxford English Dictionary* defines “appropriation” as “the assignment of anything to a special purpose” [26] In this step, we redesigned the ICF to be relevant for a wider swath of behaviors. This process consisted of three steps: (1) examining and pruning the ICF, (2) ensuring that all nodes in the hierarchy themselves represented behaviors, and (3) defining the behavior represented by each node. The lead author has over 20 years of experience with classification development and oversaw every step of ICF appropriation. No member of the team was particularly familiar with the ICF classification before the project, but it is a well-described classification and our initial expectation that it would work well for the classification of behaviors from our selected set of articles was proven correct.

**Fig 1:**
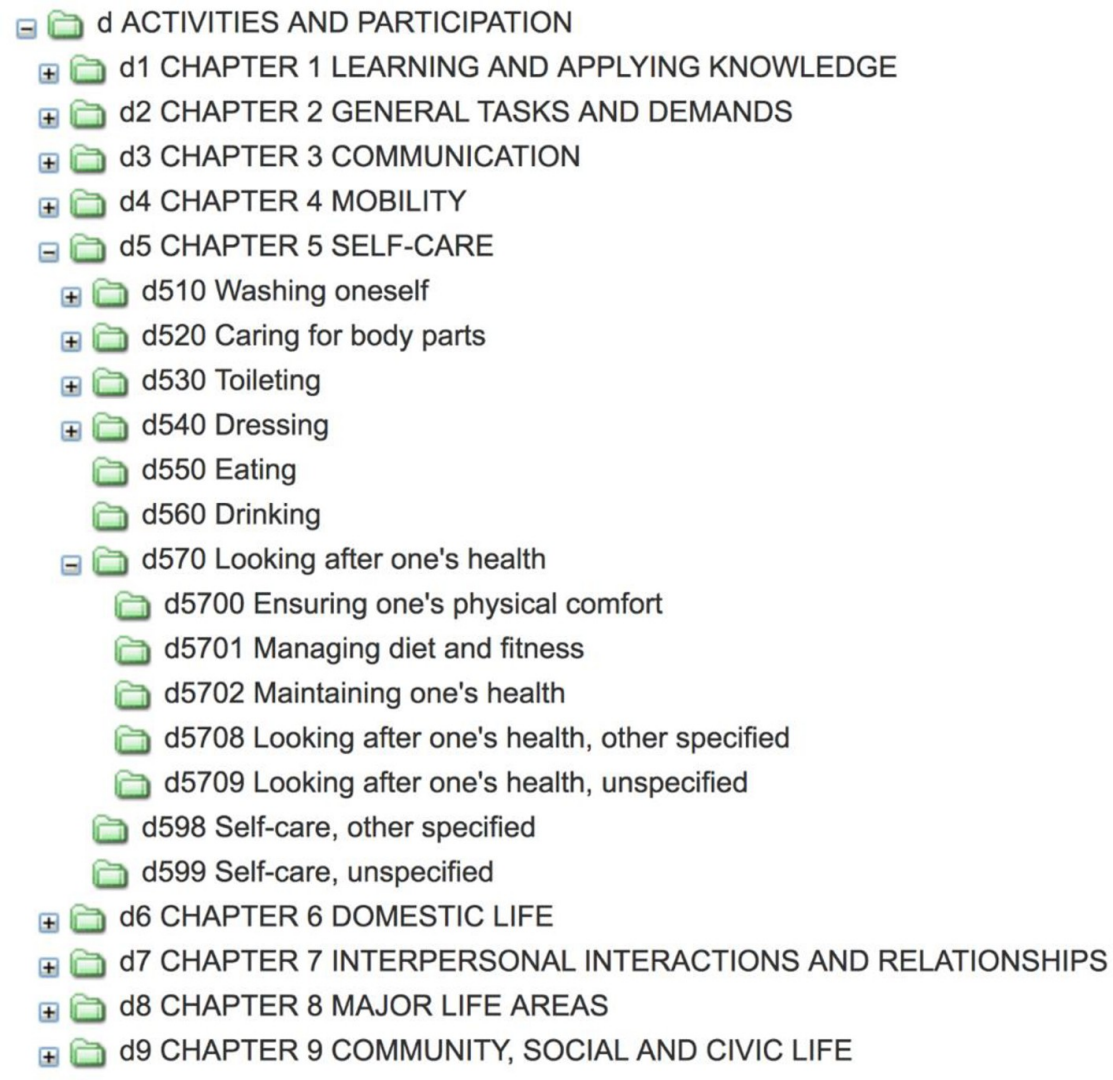
ICF category D with d570 expanded (http://apps.who.int/classifications/icfbrowser/).

1. **Examining and pruning the ICF**. Category *B*. *(Body Functions)*, *S*. *(Body Structures)*, and *E*. *(Environmental Factors)* did not fit the definition of human behaviors described previously. However, Category *D*. *(Activities and Participation)*, the only category relevant for the classification of behaviors, was selected for further examination. Categories in the ICF that were unspecific (such as “d599 Self-care, unspecified”) were removed from the hierarchy, leaving a broad framework starting at the topmost general behavior, as shown in original form in Fig 1.
  The extension of the ICF required a complete re-examination and re-evaluation of the existing hierarchy, categories, and behaviors. The fuller understanding provided by the first round of categorization enabled the examination of relationships between behaviors contextualized against the imposed relationships implicit in the ICF.
2. **Turning every level in IC-Behavior into a behavior**. For a behavior taxonomy to be complete, every node in the hierarchy should itself be a behavior. For example, ICF’s categories “Eating” and “Drinking” were kept, but we placed a new level above them named “Sustenance ingesting.” We did this to allow certain dimensions, such as the energy and the content of food, to apply to the upper level of the sustenance domain. A minimum of five experts evaluated each decision.
3. **Defining each node in the hierarchy**. While the behaviors extracted from the academic papers often had definitions, ICF categories often do not; therefore, we had to define every node in the hierarchy. For all nodes, we referenced dictionaries, the NCI Thesaurus (https://ncit.nci.nih.gov/ncitbrowser), and the NCI Metathesaurus (https://ncim.nci.nih.gov/ncimbrowser/). For future interoperability, all appropriate links to the thesauri are maintained in the taxonomy.

It is worth noting that there is an important difference between developing a taxonomy and applying that taxonomy. In our work, we carefully retained not only unique node codes, but also references to ICF codes when a node was recoded and renamed. This retention should enhance a user’s ability to use IC-Behavior to code behaviors in medical records using existing linking rules [e.g., 27].

### Step 4: Hierarchy testing and refinement

**Fig 2:**
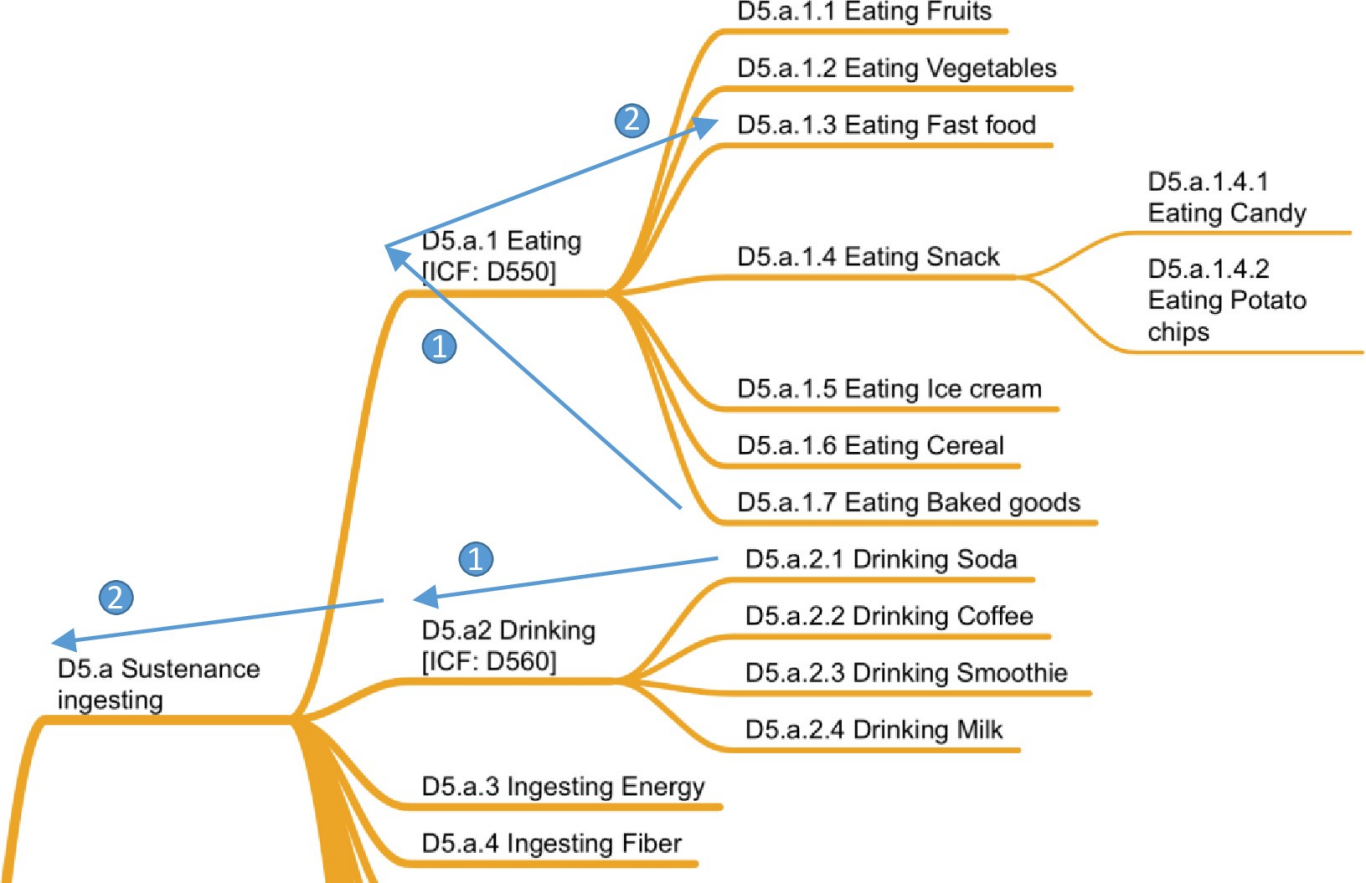
Close agreement rule.

To test the hierarchy, two researchers who had not been involved in the first stages of the project coded 1,026 previously retained behaviors into the hierarchy using specific coding guidelines (see S2 File). In each round, the two researchers independently coded a batch of 60–120 behaviors, and established final agreement through discussion. We report the pre-discussion agreement in Table 1, as well as information about how many dependent variables did not fit our operationalization of behaviors and were therefore excluded. For some behaviors, no pre-existing category existed and had to be created; these categories are separately reported in Table 1. The following rules used in this step are in line with those proposed by Worrall et al. [28] when reduced to the context of new hierarchy testing and refinement:

1. If both coders independently agreed that a variable should be removed because it did not fit the definition of a behavior, this variable was counted as an excluded variable rather than as an agreement.
2. If one coder suggested that a variable should be removed or that a new behavior node was needed in IC-Behavior, both coders discussed this concern.
  - If after discussion, the two coders agreed to the removal or request for a new node, this behavior was counted as an excluded variable.
  - If after discussion, the two coders did not agree to removal or request for a new node, this issue was counted as a disagreement between the two coders.
  - If one person did not code a variable, regardless of the outcome of the discussion, this issue was counted as a disagreement. A typical example was the case of the variable “24-hour quit attempt,” which had no provided definition. The coder with a Ph.D. in behavioral medicine classified the variable as *Quitting*, whereas the coder with a Ph.D. in business did not categorize the variable and commented, “Quit what?”
3. Two kinds of agreement were tracked. First, *agreements* were counted when both coders independently selected the same node in which to place a variable. Second, *close agreements* were recorded when the coders placed the variable within two steps of each other in the taxonomy. For example, in Fig 2, if one coder selected *Drinking milk* and the other selected *Drinking* or even *Sustenance ingesting*, this slight discrepancy would be counted as a close agreement. However, if one coder selected *Ingesting fiber* and the other selected *Eating cereal*, this significant discrepancy required traversing three steps in the taxonomy and was hence counted as a disagreement.

**Table 1:** Evaluation results.

Round	Decisions	Agreements	Close Agreements	Total agreements	New categories	Excluded variables	Total excluded
1	93	48 (51.6%)	13 (14.0%)	61 (65.6%)	12	15	27
2	91	63 (69.2%)	13 (14.2%)	76 (83.5%)	9	7	16
3	82	62 (75.6%)	11 (13.4%)	73 (89.0%)	8	14	22
4	88	63 (71.6%)	10 (11.3%)	73 (83.0%)	4	12	16
5	58	46 (59.3%)	6 (10.3%)	52 (89.7%)	1	1	2
6	96	63 (65.6%)	11 (11.5%)	74 (77.1%)	3	7	10
7	91	59 (64.8%)	7 (7.7%)	66 (72.5%)	6	7	13
8	94	74 (78.7%)	8 (8.5%)	82 (87.2%)	1	15	16
9	92	72 (78.3%)	8 (8.7%)	80 (87.0%)	3	4	7
10	99	77 (77.7%)	13 (13.1%)	90 (91.0%)	0	13	13

In the process of coding the extracted behaviors, the two researchers iteratively made refinements in the structure of the hierarchy to bolster its overall consistency and insure comprehensiveness based on (a) conceptual/expert knowledge and (b) data that did not fit, provided that the two agreed on the changes to be made. Whenever there was disagreement or a decision was difficult to make, the two coders discussed the proposed changes with the first author.

The introduced refinements included (1) adding more specific categories under a general one, (2) differentiating general categories or domains that served mere classification purposes (i.e., to aggregate different behaviors under a common node) from behavioral categories by shading domains in grey and marking behavioral categories in blue (removed from final version), (3) integrating ICF domains with behavioral categories by verbing the former, (4) ensuring consistency across the hierarchy, and (5) avoiding redundancy. *D5*.*c*.*4 doing a clinical screening* had only one subordinate behavior, i.e., *Testing*, which then split-up in two sub-behaviors, i.e., *D5*.*c*.*4*.*1 Participating in testing* and *D5*.*c*.*4*.*2 Self-testing*. In this case, we decided to delete *Testing* because (a) clinical screening is broader, and some behaviors that fall into “clinical screening” may not necessarily involve testing but (b) all testing is encompassed by “clinical screening.” (e.g., when a category at one level of the hierarchy has only one category in the next level of the hierarchy, this may indicate that one of the distinctions is not necessary). The depth and specificity of behaviors in a domain are indicative of the variety of different behaviors found in the sample of papers reviewed, rather than importance.

At the end of the refinement phase, we verified all numbering and added definitions to the newly created categories. The results of the evaluation and refinement over 10 rounds are shown in Table 1. Total agreement was 65.6% in the first round, but increased to 91.0% by the tenth round. Agreement temporarily deteriorated in rounds six and seven due to a large reorganization of the taxonomy after round five. In the last three rounds (rounds 8–10) of evaluation, only four new taxonomy nodes were added, and the coders had direct agreement rates between 77.7% and 78.7% and close agreement rates between 87.2% and 91.0%. These agreement rates are very high for a taxonomy of the complexity and size developed in this paper [29]. While it would be common to calculate Cohen’s Kappa [30] to evaluate inter-rater agreement and to remove agreement due to chance, within a framework of hundreds of available options agreement due to chance is negligible, so agreement percentage will be nearly identical to Cohen’s Kappa (if divided by 100). It is therefore uncommon to apply Cohen’s Kappa to categorization exercises at this level of complexity.

### Step 5: Taxonomy evaluation

As a final evaluation of the taxonomy, two graduate students were trained in its structure and use. They were then independently given a set of the same 100 behaviors and asked to classify them into the taxonomy. Table 2 outlines the results of this test. The results were quite encouraging in that the participants were in complete agreement 81% of the time and were in agreement 89% of the time, suggesting that the IC-Behavior taxonomy was ready for external use. We then encoded and visualized the taxonomy using MindNode (www.mindnode.com).

**Table 2:** Final evaluation agreement levels.

Group	Decisions	Agreements	Close Agreements	Total agreements	New categories	Excluded variables	Total excluded
G2	100	81 (81%)	8 (8%)	89 (89%)	0	0	0

### Step 6: Evaluation of taxonomy against past behavioral medicine taxonomies

As a final test and expansion of IC-Behavior, we collected 10 extant taxonomies or behavior frameworks from behavioral medicine (hereafter, “the 10 frameworks”) and classified them according to our taxonomy. The primary author categorized the behaviors from the 10 frameworks into IC-Behavior, and one of the coders involved in Step 4 evaluated all classifications and suggested changes as necessary. These changes were discussed and evaluated. The evaluation resulted in minor but important changes to IC-Behavior, which are outlined in S3 File.

For ease of future expansion and use, we decided to renumber the whole taxonomy; however, we kept the links (where appropriate) to the original ICF categories after the name of the behavior. A decision was made to keep the same numbers for the highest level of IC-Behavior consistent with ICF, which means that IC-Behavior is missing one high-level branch that exists in ICF (D2) but was not found useful for containing behaviors. All evaluations were done with the original numbering scheme. The upper level is *D*. *Engaging in activities/participating [ICF*: *D*]. The nine nodes below D are numbered D1–D10, starting with *D1*. *Engaging in learning and applying knowledge [ICF*: *D1]*. Initially, there were nine levels of the taxonomy, but through the discussion process in Step 4, we reduced it to six levels. The remaining four levels of IC-Behavior are coded with periods between each new level to avoid confusion in the event that numbers higher than 9 are required. Also, to make the renumbering more evident, the level under the nodes D1-D10 started with a letter (instead of a number, as in the ICF). For example, the level six node *Eating candy* is numbered D5.a.1.4.1. The third level may go between *a* and *z* if necessary, and the remaining levels may be numbered between *1* and *n*.

## Results

By the end of the development process, IC-Behavior included 253 different options for classification of behaviors, making it the largest taxonomy of behaviors developed.

Table 3 contains the first two levels of IC-Behavior with codes, names (including original ICF name when appropriate), definition, and links to NCI Thesaurus and Metathesaurus when relevant. Every level of the IC-Behavior taxonomy specifies behaviors where the leaf-nodes are very specific and clear behaviors, whereas the higher-level behaviors are more general. Nevertheless, the process of specifying which behaviors exist under a higher-level node provides implicit definitions on top of the definitions given for every node in the hierarchy.

**Table 3:** IC-Behavior top level nodes, definitions, and links to NCI Thesaurus/Metathesaurus.

Code	Name [ICF Name]	Definition	NCI Thesaurus/Metathesaurus
D.	Engaging in activities/participating [Activities and participation]	The condition in which things are happening or being done and/or the action of taking part in something (Oxford Dictionary).	C25608/C0679823
D1.	Engaging in learning and applying knowledge [Learning and applying knowledge]	The acquisition or application of knowledge or skills through study, experience, or being taught (Oxford Dictionary).	C19369/C0023185
D3.	Communicating [Communication]	The act or process of using words, sounds, signs, or behaviors to express or exchange information or to express your ideas, thoughts, feelings, etc., to someone else (Merriam-Webster).	C16452/C0009452
D4.	Moving/exercising [Mobility]	To change position or make one´s body change position in a way that can be seen, heard or felt; physical activity that someone does to stay healthy or become stronger (Merriam-Webster, adapted).	C121370/CL493908
D5.	Engaging in self-care behavior [Self-care]	The provision of what is necessary for one´s health, welfare, maintenance, and protection (Oxford Dictionary).	na/na
D6.	Engaging in domestic life activities [Domestic life]	"Carrying out domestic and everyday actions and tasks" [22, p. 153].	na/na
D7.	Engaging in interpersonal interactions and relationships [Interpersonal interactions and relationships]	The act of engaging in short or long-term association between two or more people, including kinship relations, romantic, business, and social interactions (NCI Thesaurus, adapted).	C92454/C0021797
D8.	Engaging in behavior related to major life areas [Major life areas]	Carrying out actions required to engage in education, work, and employment, and to conduct economic transactions, including the use of technologies (ICF, adapted).	na/na
D9.	Engaging in behavior related to community, social, and civic life [Community, Social, and Civic Life]	Engaging in activities related to a unified body of individuals, including recreation, community affairs, and pro/antisocial behavior (Merriam-Webster, adapted).	na/na
D10.	Engaging in mood/state changing activities and behavior	Engaging in behaviors causing changes in the conscious state of mind or predominant emotion (Merriam-Webster, adapted).	na/na

When existing in ICF, the ICF name is provided in square brackets [].

**Fig 3:**
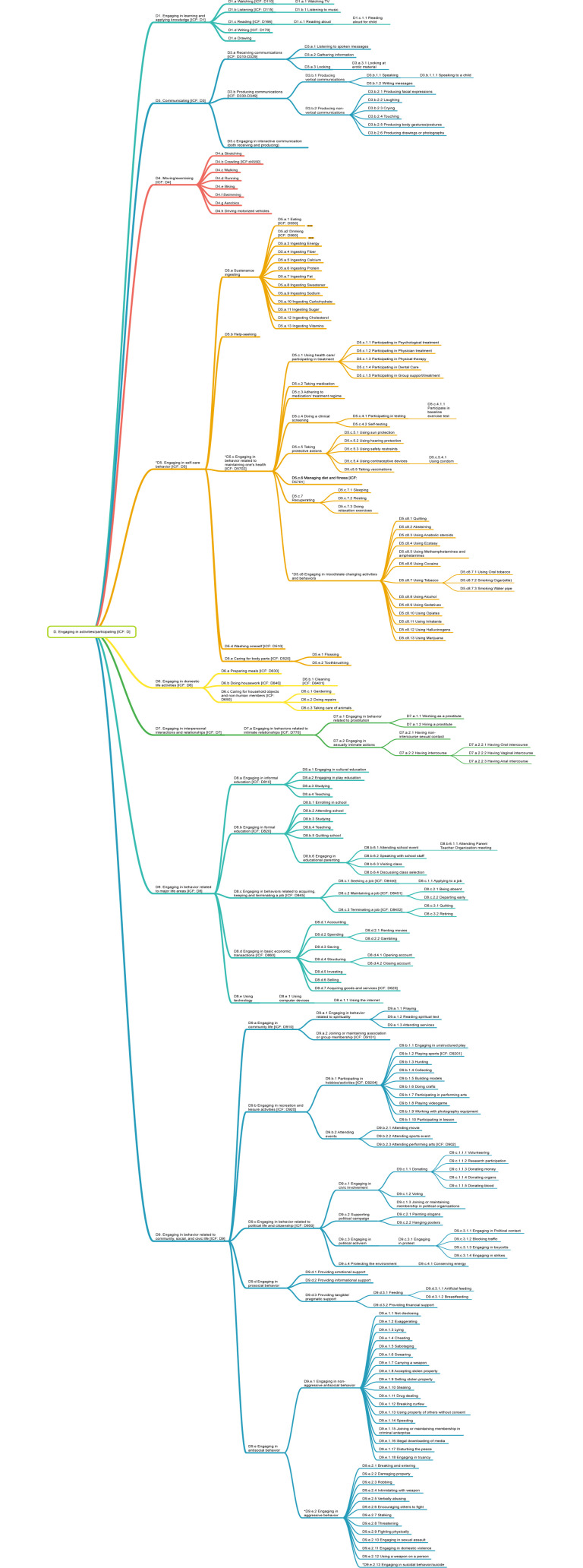
IC-Behavior taxonomy.

As may be seen from Table 3, IC-Behavior contains no D2, which appears in ICF as *General Tasks and Demands*; this node contains sub-nodes such as *undertaking a simple task*, *undertaking a complex task*, and *undertaking a single task independently*. At present, we have not encountered behaviors that are better fits within this domain than within other parts of IC-Behavior, likely due to the general nature of D2 in ICF. In ICF, these domains were likely included to address an individual’s level of functioning rather than the specific behaviors in which an individual engages. IC-Behavior is agnostic to the difficulty of a given behavior; it considers such evaluations to be context-specific and better addressed in potential future efforts to combine IC-Behavior with other ontologies. Fig 3 shows an overview of the whole ontology; a poster-size.pdf version is available at https://bit.ly/3rokNy4.

What follows is a review of the nine areas of IC-Behavior, in which we also share lessons learned from the development of the taxonomy. Because IC-Behavior is built from behaviors published as the focal point (dependent variables) of top journal articles in nine disciplines, as well as a few commonly covered behaviors in behavioral medicine taxonomies, it represents a snapshot of the behaviors deemed most important by some of the largest social and behavioral sciences.

### D1. Engaging in learning and applying knowledge [ICF: D1]

The first domain in the IC-Behavior taxonomy relates to an individual’s acquisition or application of knowledge or skills. It is tempting to think of this domain as human use of the five senses for input, but it focuses only on sight and hearing, as well as the productions of writing and drawing. The literature emphasized the extent of *watching TV* (D1.a.1), often for the purposes of examining obesity [e.g., 31] or sedentary behaviors [e.g., 32]. In terms of listening, the focus was on listening to music [e.g., 33], but one could easily imagine a combination of *listening* (D1.b) and *reading* (D1.c) in terms of using audio-books. We believe such behaviors are research-worthy given the enormous economic and learning implications; however, in this version of IC-Behavior, we only extended ICF based on available evidence. One difficult decision encountered in this domain concerned *reading aloud* (D1.c.1), which was generally focused on the contextual setting of a child being within hearing proximity, where the presence of the child would, by our definition of behavior, qualify as context. After careful consideration and realization that such reading happens specifically with the presence of the child in mind—whose presence is critical to enacting the behavior—we introduced *Reading aloud for child* (D1.c.1.1) as a specific behavior [e.g., 34]. We see this distinction as equivalent to a person sitting in a chair versus sitting (riding) on a motorcycle; it is clearly a separate behavior, even if the person on the motorcycle is merely a passenger.

**Fig 4:**
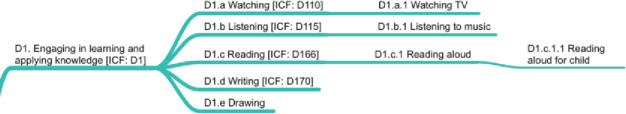
D1 domain on engaging in learning and applying knowledge.

It is worth noting a potential relationship between *engaging in learning and applying knowledge* (D1) and *engaging in informal education* (D8.a). Whereas *engaging in learning and applying knowledge* (D1) focuses on the “micro”-behaviors related to education and learning, *engaging in informal education* (D8.a) and *engaging in formal education* (D8.b) are “macro”-behaviors. In IC-Behavior, we use micro- versus macro-behaviors to refer to the existence of related behaviors and the different levels of analysis for these behaviors primarily as *part of* a relationship rather than *as a* relationship (or a *type of*). We also acknowledge that the same behavior may serve as a macro-behavior in one context and a micro-behavior in another. For example, s*tudying* (D8.b.3) may comprise *watching* (D1.a), *listening* (D1.b), *reading* (D1.c), and *writing* (D1.d) in an unpredictable order. Fig 4 shows the D1 domain.

### D3. Communicating

*Communicating* focuses on the use of words, signs, and behaviors with the intent to express ideas and thoughts to others in concert with those others. As such, *communicating* represents a complex macro-level interplay between two or more parties consisting of a stream of micro-behaviors. The *communicating* domain adheres to ICF’s split of communication into two areas focused on *receiving communications* (D3.a) and *producing communications* (D3.b). We made a difficult choice relative to *receiving communications* in accepting ICF’s highest levels, which means that whereas *listening* (D1.b) might well apply in the context of *communicating*, we maintained this organization because these micro-behaviors are split into different areas in ICF. We note, however, that when using this taxonomy for ontology-learning tasks (extraction of content from articles) and machine-learning evaluation of behavior similarities, a direct link between *Listening to spoken messages* (D3.a.1) and *listening* (D1.b) should be maintained. The behaviors *gathering information* (D3.a.2) and *looking* (D3.a.3) were retained under *receiving communications* (D3.a). One difficult choice we faced had to do with the variables that became *looking at erotic material* (D3.a.3.1) [e.g., 35]. The coding team disagreed over its eventual placement, and it could arguably have been placed under *watching* (D1.a). For future work on IC-Behavior, community input will be gathered on this issue.

**Fig 5:**
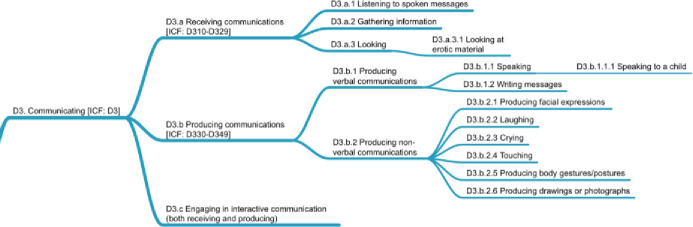
D3 domain on communicating.

*Producing communications* (D3.b) was split into two parts, focusing on verbal versus non-verbal communication. *Producing verbal communications* (D3.b.1) focuses on speaking and writing messages, whereas *producing non-verbal communications* (D3.b.2) contains a number of categories related to *facial expressions* (D3.b.2.1), *laughing* (D3.b.2.2), *crying* (D3.b.2.3), *touching* (D3.b.2.4), and *body postures* (D3.b.2.5), as well as *production of drawings and photographs* (D3.b.2.6). The production of drawings and photographs is seen as a subtle way of communicating through the selection of themes and foci. Fig 5 shows the D3 domain.

### D4. Moving/Exercising

**Fig 6:**
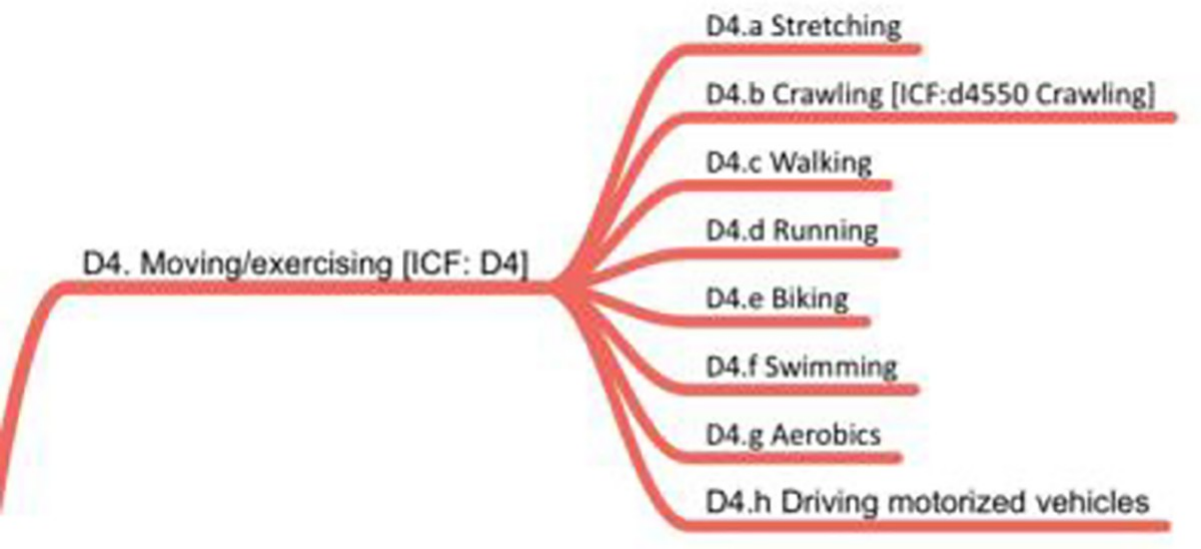
D4 domain on moving/exercising.

*Moving/exercising* focuses on intentional movement—that is, changes of position or body movements that are significant enough to be seen, heard, or felt. What makes these moves different from many other body movements in the taxonomy is the emphasis on the underlying intent to stay healthy, become stronger, or provide transport. Such movement includes *stretching* (D4.a), *walking* (D4.c), *running* (D4.d), *biking* (D4.e), *swimming* (D4.f), and *aerobics* (D4.g). One surprise in this domain is *crawling* (D4.b), which was kept for backward compatibility with ICF although it was not encountered as a research-worthy behavior in our dataset. Nevertheless, for very young human beings, crawling is absolutely a paramount behavior. The biggest outlier in this domain may well be *driving motorized vehicles* (D4.h); while our coders agreed that it fit best at this taxonomic location, its placement may be reconsidered in future versions. Fig 6 shows the D4 domain.

### D5. Engaging in self-care behavior

Self-care behaviors are defined as those human behaviors that are necessary for one’s health, welfare, maintenance, and protection. At the top of D5, *sustenance ingesting* (D5.a) represented an interesting challenge in that there were specific examples of eating and drinking, even though these must be seen as limited exemplars. Use of the taxonomy in this area could profitably be combined with existing food ontologies or databases, such as the USDA National Nutrient Database for Standard Reference [36] or the German food database fddb.info [one use of which is reported in 37]. Adding sustenance ingesting to this domain proved to be a difficult decision, given that IC-Behavior focuses on actual behaviors and many articles reported sustenance ingesting by emphasizing the nutritional components of food eaten (e.g., *energy*, *fiber*, *calcium*, *protein*). Because variables were clearly calculated based on reported behaviors, we decided to make these variables part of the D5.a behavior as D5.a.3–D5.a.10.

*Help-seeking* (D5.b) refers to a person requesting assistance with a task, job, or problem. This category was employed for general help-seeking in all aspects of life that is unrelated to the use of health-care services, which are covered by *engaging in behavior related to maintaining one’s health* (D5.c); the split was necessary in order to maintain as much backward compatibility with ICF as possible. *D5*.*c* consists of seven immediate branches, starting with *D5*.*c*.*1 Using health care/participating in treatment*, which covers participation in treatment by a physician or dental professional, individual or group treatment for psychological conditions, and receiving physical therapy.

*Taking medication* (D5.c.2), a micro-behavior, specifically focuses on whether a given medication is taken on a one-time basis. The related behavior, *adhering to medication/treatment regime* (D5.c3), is a macro-behavior: it relates to all the categories of D5.c.1 because the medication/treatment stemmed from one of the D5.c.1 behaviors. That said, this behavior also relates to D5.c.2 because of its focus on following a provider’s treatment recommendation as a longitudinal process with respect to timing, dosage, and frequency over a specified length of time. *Doing a clinical screening* (D5.c.4) includes both participation in testing and self-testing, as self-tests have become more common. This hierarchy could well expand indefinitely over time; therefore, we recommend future work with this part of the taxonomy include expansions of the hierarchy by employing other taxonomies or ontologies that specify types of tests and their relationships.

*Taking protective actions* (D5.c.5) centers on covering or shielding oneself from risks to maintain one’s health. Perhaps the most difficult decision here was to place *using condom* (D5.c.5.4.1) under *Using contraceptive devices* (D5.c.5.4), because a condom has additional functions beyond serving as a contraceptive as well as a clear connection to *engaging in behaviors related to intimate relationships* (D7.a).

*Managing diet and fitness* (D5.c.6) focuses on adjusting eating, drinking, and exercise according to prescribed rules for the purpose of improving or maintaining health. It has connections to *sustenance ingesting* (D5.a) and *moving/exercising* (D4). Finally, *recuperating* (D5.c.7) focuses on relaxation exercises and rest, including *sleeping* (D5.c.7.1). In this domain, it is worth highlighting the *sleeping* category. In ICF, *sleeping* belongs in the B-tree as “b134 Sleep functions.” While we recognize that sleep itself may not be a behavior, it has immense implications on behavior [38,39]; therefore, we listed it as a behavior. This decision aligns with those made in 7 of the 10 behavior frameworks produced in behavioral medicine.

An important subdomain (D5.c8) focuses on *engaging in mood/state changing activities and behaviors*, those behaviors that cause changes in the conscious state of mind or predominant emotion. In addition to the use of a variety of drugs such as *anabolic steroids* (D5.c8.3), *cocaine* (D5.c8.6), and *tobacco* (D5.c8.7), this domain contains behaviors related to *abstaining* (D5.c8.2) and *quitting* (D5.c8.1) for each type of drug, because of the surveyed literature’s heavy emphasis on these behaviors. As discussed earlier, an ontology of behaviors should consider the different types of behaviors and establish which behaviors are amenable to quitting and abstaining. The final meta-level behavior was *engaging in suicidal behavior/suicide*—a grim end to this taxonomy and certainly one for which *abstaining* has meaning.

**Fig 7:**
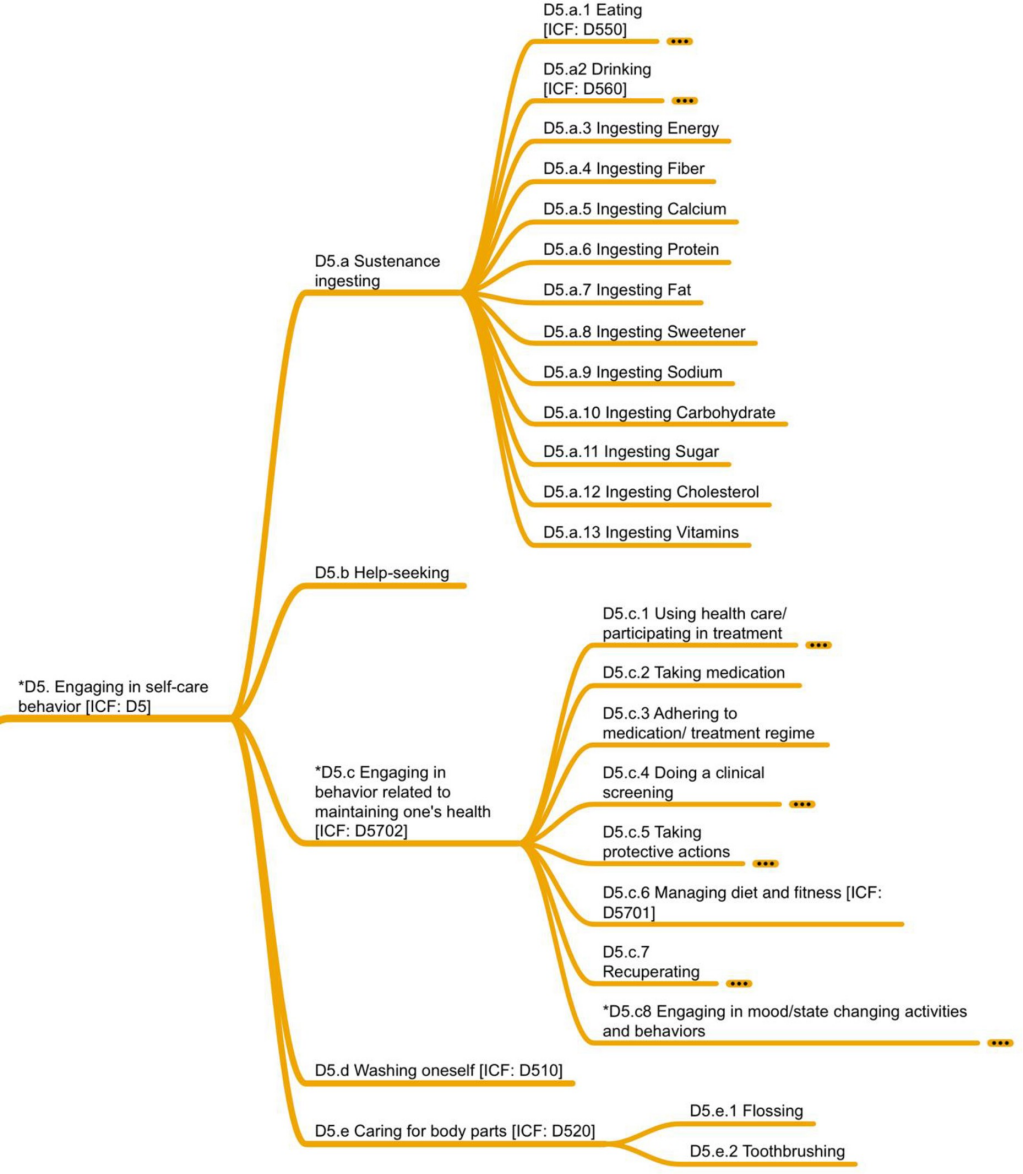
D5 domain on engaging in self-care behavior.

Finally, the D5 domain finishes with *washing oneself* (D5.d) and *caring for body parts* (D5.e); these behaviors are kept separate to maintain backward compatibility with ICF. In terms of caring for body parts, only two types were found in the reviewed literature: *flossing* (D5.e.1) and *toothbrushing* (D5.e.2). While one may imagine many other body parts as worthy of care and even of great economic significance (e.g., nails), for various reasons, these may not have reached the threshold of significance often associated with work published in top journals. Fig 7 shows the D5 domain in a compressed fashion. For the whole hierarchy, see the poster-size version.

### D6. Engaging in domestic life activities

**Fig 8:**
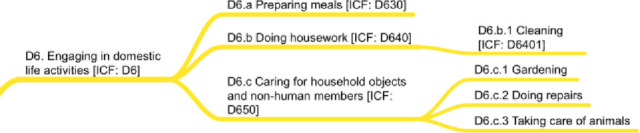
D6 domain on engaging in domestic life activities.

One of the largest areas of human activity, “carrying out domestic and everyday actions and tasks" [22], is also one of the smallest domains of IC-Behavior. This area affects well-being, diet, and exercise, but has not itself been the focus of extensive research. Foci include *preparing meals* (D6.a), which brings better control over one’s diet and as such is related to *sustenance ingesting* (D5.a). *Doing housework* (D6.b) allows a person to move around and reduce clutter and messes, behaviors highlighted in extant behavioral medicine frameworks [i.e., 4,13]. Finally, *caring for household objects and non-human members* (D6.c) centers on taking care of a house or apartment, as well as any household animals. Fig 8 shows the D6 domain.

### D7. Engaging in interpersonal interactions and relationships

This domain focuses on acts of engaging in short- or long-term association between two or more people, including kinship relations and romantic, business, and social interactions (definition adapted from NCI Thesaurus). While this domain was set up to accept additional types of relationship behaviors, only *engaging in behaviors related to intimate relationships* (D7.a) was encountered in the literature. This type of relationship behavior split into *engaging in behaviors related to prostitution* (D7.a.1), either as a prostitute or as a person hiring a prostitute.

**Fig 9:**
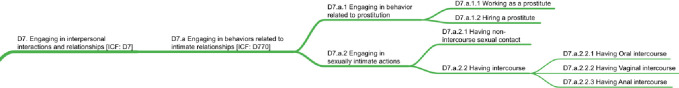
D7 domain on engaging in interpersonal interactions and relationships.

The coders struggled quite a bit with placement of *engaging in sexually intimate actions* (D7.a.2.2), where research interest in the macro-behavior of *having intercourse* (D7.a.2.2) split between *oral* (D7.a.2.2.1), *vaginal* (D7.a.2.2.2), and *anal* (D7.a.2.2.3) intercourse. Initially, the coders placed *using contraceptive devices* (coded as D5.c.5.4)—especially *using condom* (D5.c.5.4.1)—with the intercourse behaviors, which resulted in separate behaviors such as having vaginal intercourse with condom. If the combination of behaviors is important in coding, users of IC-Behavior are advised to code this behavior as D7.a.2.2.2 (having vaginal intercourse) and D5.c.5.4.1 (using condom). Fig 9 shows the D7 domain.

### D8. Engaging in behavior related to major life areas

**Fig 10:**
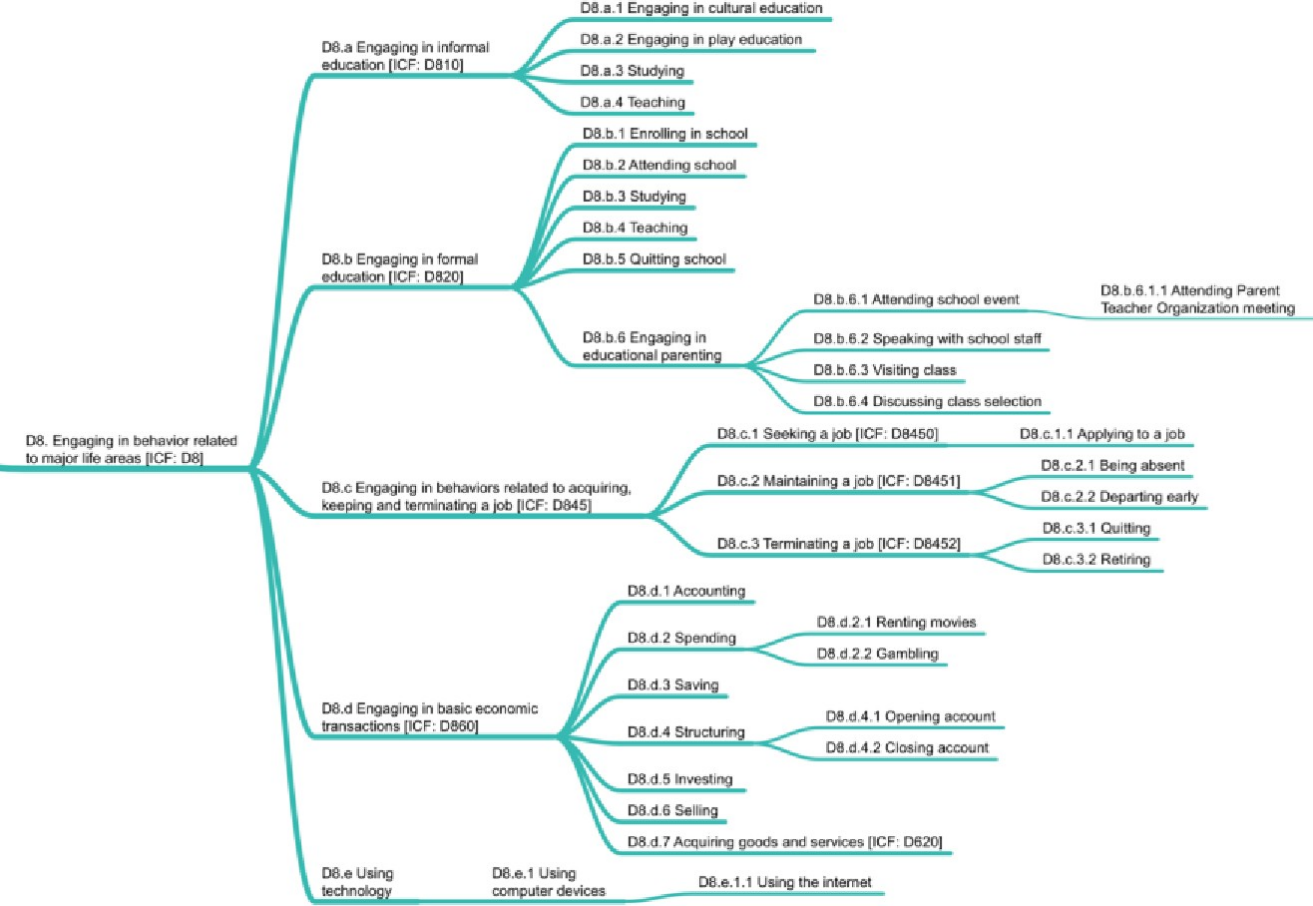
D8 domain on engaging in behavior related to major life areas.

This domain includes behaviors related to carrying out actions needed to engage in education, work, and employment and to conduct economic transactions, including technology use. To remain consistent with ICF, we distinguished between *engaging in informal education* (D8.1), which specifically focuses on *engaging in cultural education* (D8.a.1), and *engaging in play education* (D8.a.2). In addition, *studying* (D8.a.3) and *teaching* (D8.a.4) exist as informal educational behaviors as well as as behaviors in *engaging in formal education* (D8.b; see D8.b.3 and D8.b.4). Other behaviors related to formal education are *enrolling in school* (D8.b.1), *attending school* (D8.b.2), and *quitting school* (D8.b.5), as well as *engaging in educational parenting* (D8.b.6). While the goal for IC-Behavior was to focus on the behavior rather than the context in which it takes place, the non-interactional nature of a hierarchy forced a separation of *attending parent teacher organization meeting* (D8.b.6.1.1) from such behaviors as *attending* (*religious*) *services* (D9.a.1.3) and *attending events* (D9.b.2). Fig 10 shows the D8 domain.

*Engaging in behaviors related to acquiring*, *keeping*, *and terminating a job* (D8.c) addresses job behaviors, starting with *seeking a job* (D8.c.1). Given the diversity, number, and importance of micro-behaviors that exist under *maintaining a job* (D8.c.2), future work on IC-Behavior should expand this category.

The node related to *engaging in basic economic transactions* (D8.d) allows the tracking of money use, specifically including *spending* (D8.d.2) and *acquiring goods and services* (D8.d.7); maintaining or improving financial status through *saving* (D8.d.3), *investing* (D8.d.5), or *selling* (D8.d.6); or organizing finances through *accounting* (D8.d.1) and *structuring* (D8.d.4). Finally, *using technology* (D8.e) was set up to facilitate future expansion and thereby encompass more micro-behaviors for specific technologies. *Using the internet* (D8.e.1.1) was placed under *using computer devices* (D8.e.1) because the internet is not generally usable other than through computing devices—even if a device can seem useless without internet access.

### D9. Engaging in behavior related to community, social, and civic life

**Fig 11:**
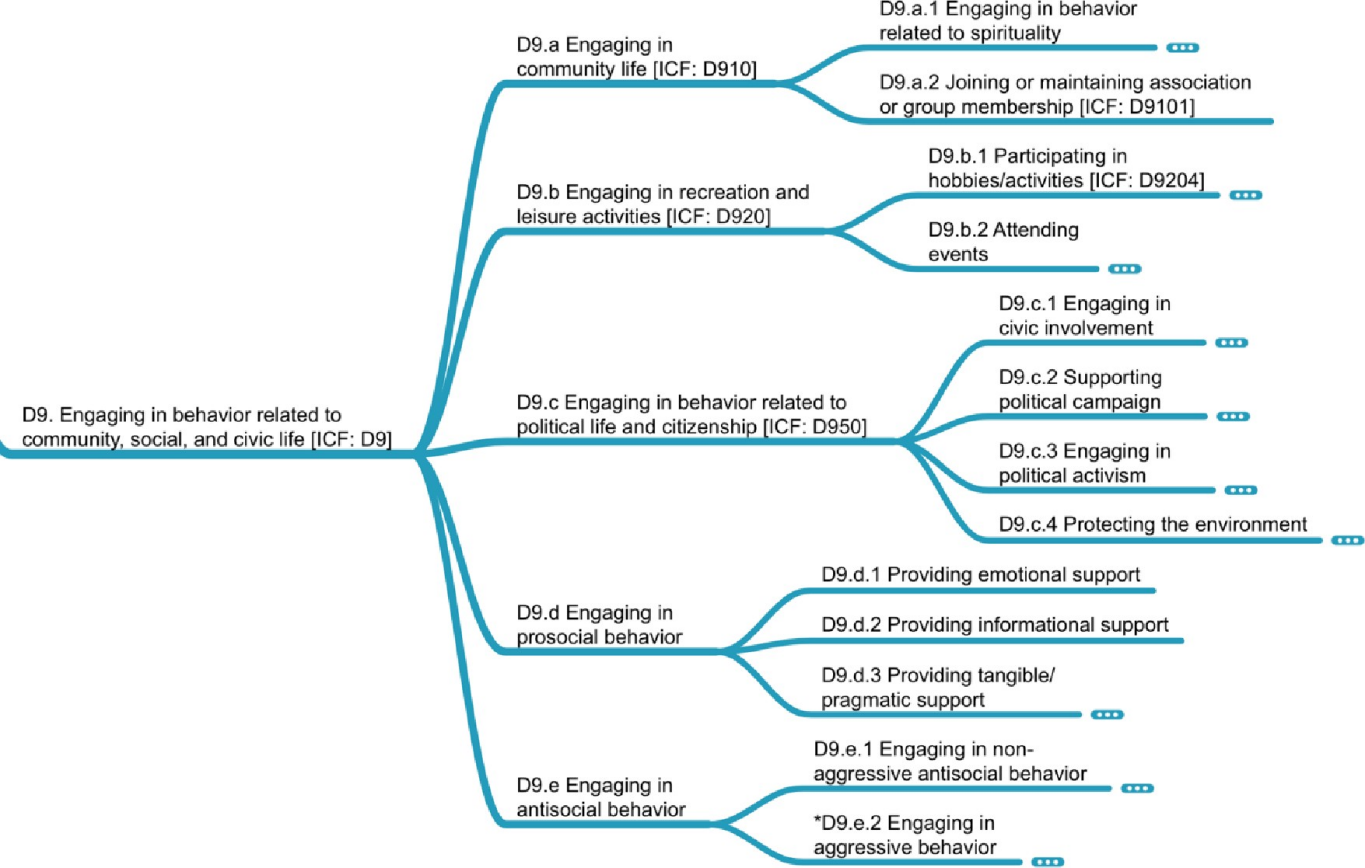
D9 domain on engaging in behavior related to community, social, and civic life.

Fig 11 shows a part of this domain, which was defined as “engaging in activities related to a unified body of individuals, including recreation, community affairs, and pro/antisocial behavior.” When *engaging in community life* (D9.a), the common examples found included *engaging in behavior related to spirituality* (D9.a.1) and *joining and maintaining association or group membership* (D9.a.2), which was not further detailed in our data. We encountered a more fully developed node under *engaging in recreation and leisure activities* (D9.b), which split into *participating in hobbies/activities* (D9.b.1) and *attending events*. Both contain behaviors of great economic and recuperative significance.

*Engaging in behaviors related to political life and citizenship* (D9.c) was found to contain four macro-behaviors, beginning with *engaging in civic involvement* (D9.c.1). This macro-behavior included *donating* (D9.c.1.1) time through *volunteering* (D9.c.1.1.1) or *research participation* (D9.c.1.1.2), as well as donating *money* (D9.c.1.1.3), *organs* (D9.c.1.1.4), and *blood* (D9.c.1.1.5). Similarly, this civic involvement encompasses *voting* (D9.c.1.2) and *joining or maintaining membership in political organizations* (D9.c.1.3). The second macro-behavior indicated a more specific interest in an issue or candidate—*supporting a political campaign* (D9.c.2) through specific behaviors—which would almost certainly include *donating money* (D9.c.1.1.3). The third signaled even greater engagement: *engaging in political activism* (D9.c.3) may include *protest* (D9.c.3.1), even at the risk of one’s own health and economic future. Finally, such civic engagement may include behaviors related to *protecting the environment* (D9.c.4).

The final two areas discovered for this domain focus on *engaging in prosocial behavior* (D9.d); for example, by *providing emotional support* (D9.d.1), *providing informational support* (D9.d.2), and *providing tangible/pragmatic support* (D9.d.3). The second area, *engaging in antisocial behavior* (D9.e), was split into *engaging in non-aggressive antisocial behavior* (D9.e.1), such as *stealing* (D9.e.1.10) and *drug dealing* (D9.e.1.11), and *engaging in aggressive behavior* (D9.e.2) like *breaking and entering* (D9.e.2.1), *threatening* (D9.e.2.8), and *using a weapon on a person* (D9.e.2.12).

## Discussion

This behavior taxonomy developmental study used an interdisciplinary set of behaviors and an existing taxonomy that organized a set of behaviors; the WHO’s *International Classification of Functioning Disability and Health* (ICF). The goals of ICF are broader than our goals for this taxonomy. We retained all but one (D2) of ICF’s nine domains. Thus, there are eight behavior domains: *D1*. *Engaging in learning and applying knowledge [ICF*: *D1]*, *D3*. *Communicating [ICF*: *D3]*, *D4*. *Moving/exercising [ICF*: *D4]*, *D5*. *Engaging in self-care behavior [ICF*: *D5]*, *D6*. *Engaging in domestic life activities [ICF*: *D6]*, *D7*. *Engaging in interpersonal interactions and relationships [ICF*: *D7]*, *D8*. *Engaging in behavior related to major life areas [ICF*: *D8]*, *D9*. *Engaging in behavior related to community*, *social*, *and civic life [ICF*: *D9]*. The key advantages of the framework are as follows:

1. It builds on a carefully developed and internationally adopted framework that was officially endorsed in 2001 by all 191 WHO Member States as the standard for description and classification of health and disability.
2. The framework is built (and expanded) upon behaviors that have functioned as dependent variables in top journals from nine disciplines, and thus suggests behaviors that are, on average, considered important across disciplines. As such, it may represent the first truly comprehensively developed behavior taxonomy.
3. It has been validated with high inter-rater agreement by raters not previously familiar with the taxonomy and tested against previously published behavior taxonomies.

While the process and evaluation of IC-Behavior’s final framework suggests a strong foundation, it is no longer a classification of functioning. The taxonomy may also be limited by the lack of coverage of the public health field. However, the evaluation against a large set of existing health behavior taxonomies suggests that this may not be the case. The taxonomy holds potential for expansion and refinement through future consensus studies. As currently presented, the framework should be ready for future work on the “components” of behaviors as well as considerations of these components in relation to an ontology of behaviors. IC-Behavior v. 2.0 should include information on antonyms, and whether a given behavior is considered positive or negative health-wise. It would also be useful to indicate connections between behaviors that are highly related but part of different taxonomic branches.

There are several benefits to the development of an interdisciplinary taxonomy of behaviors. First, many behaviors are examined by multiple disciplines. For example, adoption of innovations and technology is a topic covered by almost every discipline, but primarily Psychology and IS. A taxonomy provides the opportunity to establish shared definitions and language across disciplines, enabling interdisciplinary coordination and integration. Second, theories of behavior [e.g., 40] have established that the nomological networks of related behaviors are often identical. It is therefore incumbent on researchers to identify behaviors similar enough that the same nomological networks likely affect both. Such identification enables direct knowledge transfer across disciplines and theories. Finally, behavior change interventions are often evaluated in relation to specific behaviors, so by integrating this taxonomy with the Behavior Change Technique Taxonomy [41], knowledge about behavior change techniques may be extended across behaviors and disciplines.

The aim of this project was to develop a taxonomy of behaviors that are relevant for research across scientific disciplines. This goal reflects a commitment to using a shared language for referring to domain entities through knowledge sharing and reuse (Gruber, 1993). By organizing knowledge about known behaviors, we can specify which disciplines focus on the same or related behaviors, enabling better sharing of behavior-relevant research. Knowing how similar behaviors are is a first step in generalizing a nomological network from one behavior to a related behavior, thereby greatly expanding the value and reuse of knowledge.

## Conclusions

Through a six-step development and validation process, we developed an interdisciplinary ICF-linked behavior taxonomy containing eight domains, 45 intermediate higher-level behaviors and 191 leaf-level behaviors for a total of 250 documented behaviors, each carefully defined and linked to ontologies such as the NCI Metathesaurus. By building IC-Behavior, we respond directly to the Wade and Halligan’s [42, p. 349] call to prepare the WHO ICF to “be used as a powerful analytic and explanatory model of human experience and behaviour in any situation, not only in illness and disease,” but rather than build our work into ICF, we propose it as a separate classification. We believe that IC-Behavior will prove to be a useful tool for those aiming to expand and deepen description of—and investigation into—behaviors.

## Supporting information

S1 File Included journals by discipline. (DOCX)

S2 File Coding guidelines. (DOCX)

S3 File Past behavioral medicine frameworks. (DOCX)

S4 File IC-Behavior taxonomy with definitions, and thesaurus links. (DOCX)

S5 File Principal component analysis of past behavior frameworks. (DOCX)
