# Supplementary material for: IC-Behavior: An interdisciplinary taxonomy of behaviors
Source: PLoS One. 2021 Sep 17;16(9):e0252003. doi: 10.1371/journal.pone.0252003 (PMC8448352; doi:10.1371/journal.pone.0252003)
Supplement: S1 File — (DOCX) [file pone.0252003.s001.docx]

**S1 File. Included Journals by Discipline**

The journals were selected by HBP after interviews with experts of the disciplines. The included journals should reflect the best journals in each discipline, to the best understanding of those experts.

| **Field** | **Journal name** | **Year range** | **Number of included articles** |
| --- | --- | --- | --- |
| Behavioral Medicine | Annals of Behavioral Medicine | 2007-2011 | 235 |
|  | Health Education Research | 2000-2009 | 336 |
| Education | American Educational Research Journal | 1988-2007 | 143 |
|  | Sociology of Education | 2000-2009 | 66 |
|  | Educational Evaluation and Policy Analysis | 2000-2009 | 35 |
| Management | Academy of Management Journal | 2002-2011 | 271 |
|  | Administrative Science Quarterly | 2001-2010 | 42 |
| Marketing | Journal of Consumer Research | 2001-2009 | 469 |
|  | Journal of Marketing Research | 2000-2009 | 272 |
| Information Systems | Journal of Management Information Systems | 1990-2012 | 327 |
|  | MIS Quarterly | 1980-2012 | 290 |
|  | Information Systems Research | 1990-2012 | 183 |
| Nursing | Research in Nursing and Health | 2000-2009 | 290 |
|  | Nursing Research | 2000-2009 | 279 |
|  | Journal of Nursing Scholarship | 2000-2009 | 202 |
| Psychology | Journal of Applied Psychology | 1998-2011 | 930 |
|  | Journal of Personality and Social Psychology | 2005-2012 | 849 |
| Sociology | Social Forces | 2000-2009 | 230 |
|  | American Sociological Review | 2000-2009 | 119 |
|  | American Journal of Sociology | 2000-2009 | 76 |
