# Supplementary material for: IC-Behavior: An interdisciplinary taxonomy of behaviors
Source: PLoS One. 2021 Sep 17;16(9):e0252003. doi: 10.1371/journal.pone.0252003 (PMC8448352; doi:10.1371/journal.pone.0252003)
Supplement: S2 File — (DOCX) [file pone.0252003.s002.docx]

**S2 File. Coding guidelines**

**Definition of behavior:** Behaviors must be observable. For example, “spending” is a behavior; “impulsive spending” is not different from spending because, in most settings, it is not possible for an observer to distinguish between spending and impulsive spending. A behavior can have dimensions (e.g., reading for a child is a dimension of “reading,” as is reading the Bible). Dimensions can change the outcome of the behavior in significant ways, but they are not themselves behaviors. In some notable cases, such as reading a holy scripture, the behavior of reading may have special meaning. We included this type of reading, therefore, as a separate behavior. The same was true in the case of *reading aloud* versus *reading aloud for a child*.

Specific considerations of behaviors:

1. When the definition seems too broad, or we are unsure where to classify the behavior, we should look at the original paper from where the variable has been extracted.
2. Behaviors can vary in terms of their frequencies, but the frequency of a behavior should not be used to define a behavior.
3. Looking at the verb in the definition is a good way of finding what the action was.
4. That said, some variables may be too broad. We define “too broad” as the occasions when a number of behaviors exists in different places of the behavior-hierarchy such that it is not possible to code them as behaviors. Often, the particular behaviors involved are not specified, or the interpretation of it as a behavior would likely lead to low inter-rater agreement. For example, variable 16791 “symbolic response” (parent: teachers reply to policy; definition: “making changes in the appearance but not the substance of their work”).
5. When it makes sense to have a more specific category under a more general one, we can create a new category.
6. One should primarily code based on the definition (and not variable name). For example, with variable 33853 “use of tobacco or alcohol,” two behaviors are defined, but the definition only refers to alcohol intake. This decision is made only after the original paper has been re-examined to confirm that the variable could not be split up and whether the definition is correctly reported.
7. When a definition is not provided, we should code using the variable name (Column C). In these cases, because of the lack of clarity of the variable, new categories in the hierarchy should not be created.
8. One variable should only refer to one behavior. Whenever more than one behavior appears in the definition (i.e., the definition includes more than one behavior, or a general definition provides one or more examples of behaviors), then an extra row should be added with the same variable ID, and the original definition should be split among the two (or more rows) so that each row is only coding one behavior. Also, we should put what is not being considered in brackets. The same applies when more than one category could be attributed to the definition. For example, if the behavior was defined as “eating high-fiber cereals,” we would split it into two rows and code these as “D545.a8 Cereal” and “D545.c31 Fiber.”
9. When the definition provides specific examples of the general category, we should code based on the specific examples that are provided. For example, with “Adolescents’ self-reports of their participation in 14 different delinquency activities including pulling a knife/gun on someone in the past year,” we code for “pulling a knife/gun on someone” and not the general category “delinquency.”

Specific considerations about coding:

1. It is important to ask ourselves, “Can we figure out the manifest behavior based on the description provided?” For example, for variable 67810 (“Surprise choice of reward,” with the definition, “Participant's choice of the congruent reward [a free coffee] at the time they were approached”), we decided not to code this as a behavior. The reason for our decision not to code this as a behavior is that “choice” is a more cognitive process that may have a behavioral component (e.g., picking a card/pressing a button/ticking an option on a questionnaire, etc.). In other words, we do not know what the manifest part of this choice was.
2. The semantical reverse of a behavior (e.g., “quitting school”) should not be coded under its opposite category (e.g., “enrolling in school.” Neither should “abstinence” (from tobacco) be coded under “Using Tobacco,” as these categories correspond to different behaviors. The exception to this rule is when the focus of the study was the presence versus the absence of a particular behavior. For example, “not using condom” may be coded as “using condom,” as this reflects a 1/0 binary of participating/not participating in the same behavior.
3. One must code the behavior as specifically as possible (this means going down in the hierarchy as much as possible), but it is not necessary to use only the last level of each branch of the hierarchy. Behaviors may be stored at any level of the hierarchy.

When coding, we should go with the “what” and not with the “how.” For example, for the following definition, “The extent to which the support provider behaved in a dominating manner in his or her attempts to provide support,” we should focus on the provision of support (“what”) and not on the manner in which the support was provided (“how”). The focus here shifts from what kind and the intensity of support provided to *whether* support was provided.
