# Supplementary material for: IC-Behavior: An interdisciplinary taxonomy of behaviors
Source: PLoS One. 2021 Sep 17;16(9):e0252003. doi: 10.1371/journal.pone.0252003 (PMC8448352; doi:10.1371/journal.pone.0252003)
Supplement: S3 File — (DOCX) [file pone.0252003.s003.docx]

**S3 File. Past Behavioral Medicine Frameworks**

A literature review discovered 10 past behavior frameworks in the area of behavioral medicine. While evaluating these frameworks, some purported behaviors were removed because they did not fit our definition of a behavior. These removed behaviors are listed below along with a reference to the originating paper, each listed under our reason for excluding them (some could reasonably be categorized in several of these groups):

Not a unitary behavior (consists of many behaviors):

- Raising animals [1]
- Raising children [1]
- Finding an organized and orderly workplace [1]

Unobservable as specific behavior:

- Reaching a state of wholeness/peace with oneself [1]
- Making an effort to think positively [1]
- Finding meaning and purpose in life [1]
- Developing self-awareness/noticing changes in one’s body [1]
- Avoiding undue stress/Stress management/Reducing stress/Working with a balanced/moderate amount of stress [2-5]

Behavior requires subjective evaluation:

- Wearing comfortable clothes and/or shoes appropriate to the season [1]
- Doing activities one likes [1]
- Avoiding activities that might be dangerous (carrying too much weight, etc.) [1]
- Avoiding violence [1]
- Taking more chances than the average person [6]
- Avoiding high crime areas [6]

A total of 238 behaviors from the 10 frameworks were categorized into 105 unique instances of behaviors. All behavior-describing texts from the frameworks, as well as the categorization decisions, are available below. Because classification under IC-Behavior allowed us to classify the behaviors in the 10 frameworks into one 10 (frameworks) x 105 (behaviors) matrix, it was possible to conduct a principal-components analysis to better understand the extent to which any one of these past frameworks could already serve as an integrative taxonomy. Using Principal Components Analysis, varimax rotations, and extracting any principal component with an eigenvalue greater than 1 and suppressing any coefficient with an absolute value below .4, results showed that three different components of behavior exist in the 10 frameworks (61.8% of variance extracted). The PCA passed the Kaier-Meyer-Olkin Measure of Sampling Adequacy with a score of .564, suggesting that data was “suitable” for PCA [7]. The principal components are listed in S5 File{. This result is indicative of the clear need for a taxonomy to integrate disparate frameworks. We therefore added the behaviors listed in at least three past frameworks that were not already in IC-Behavior (like all behaviors that did not fit directly into IC-Behavior, these five behaviors did fit directly below an existing category):

- *Ingesting Vitamins* – included in 7 frameworks
- *Ingesting Cholesterol* – included in 4 frameworks
- *Ingesting Sugar* – included in 4 frameworks
- *Participating in Baseline Exercise Test* – included in 3 frameworks
- *Taking Vaccinations* – included in 3 frameworks

In seven cases out of 105, two different categories of the IC-Behavior were needed to categorize behaviors correctly from the 10 frameworks. The most frequent case of this multi-category issue was that the IC-Behavior does not distinguish between measurement of doing a behavior and not doing a behavior other than in a few cases of addiction-related behaviors, such as *abstaining* (IC-Behavior node D10.b) from *using alcohol* (D10.h) or *abstaining* (D10.b) from *engaging in mood/state-changing activities and behavior* (D10). Similarly, *quitting* (D10.a) *using tobacco* (D10.g), or *driving motorized vehicles* (D4.h), *biking* (D4.e), and *swimming* (D4.f) while *using alcohol* (D10.h) were coded as two behaviors occurring simultaneously. This situation may be seen as an indication of the importance of transforming the IC-Behavior to an ontology where such purposeful non-behaviors as quitting and abstaining may be set up to apply to any behavior, such as *ingesting fat* or *ingesting sodium*, rather than to alcohol and drugs alone. The final overview of the 10 frameworks organized within IC-Behavior is available in the following table.

| IC-Behavior Category | IC-Behavior 2^nd^ category | # Beh-aviors | Direct fit (D/S/N)^a^ | [8] | [9] | [2] | [10] | [3] | [4] | [5] | [11] | [6] | [1] |
| --- | --- | --- | --- | --- | --- | --- | --- | --- | --- | --- | --- | --- | --- |
| D1. Engaging in learning and applying knowledge |  | 1 | D |  |  |  |  |  |  |  |  |  | Learning and expanding one’s horizons |
| D1.a.1 Watching TV |  | 1 | D |  |  |  |  |  |  |  |  |  | Not watching a lot of television |
| D3.a.2 Gathering information |  | 5 | D |  |  | Getting information to recognize early disease signs |  | Knowledge about drug contents | Health magazines | Be knowledgeable about drug content and side effects |  | I gather information on things that affect my health by watching television and reading books, newspapers, or magazine articles |  |
| D3.b.2.2 Laughing |  | 1 | D |  |  |  |  |  |  |  |  |  | **Laughing**, having fun and being happy |
| D4. Moving/exercising |  | 8 | D | Engaging in recommended levels of physical activity / Exercising regularly | Regular physical activity | Aerobic activity three times a week |  | Aerobic activity 3 times/week |  | Engage in aerobic activity 3 or more times a week | Exercise over last 14 days | I exercise to stay healthy | Exercising |
| D4. Moving/exercising |  | 2 | S |  |  |  |  |  | Yoga |  |  |  | Engaging in Oriental arts (**yoga**, meditation, etc.) |
| D4. Moving/exercising |  | 1 | S |  |  |  |  |  | Weights |  |  |  |  |
| D4.c Walking |  | 2 | D |  |  |  |  |  | Walking |  |  |  | **Outdoor hiking** and being in nature |
| D4.e Biking | D10.h Using Alcohol | 1 | D |  |  |  | Bicycling when drunk |  |  |  |  |  |  |
| D4.e Biking |  | 1 | D |  |  |  | Bicycling -- steep downhill |  |  |  |  |  |  |
| D4.f Swimming | D10.h Using Alcohol | 1 | D |  |  |  | Swimming when drunk |  |  |  |  |  |  |
| D4.f Swimming |  | 1 | D |  |  |  |  |  | Swimming |  |  |  |  |
| D4.h Driving motorized vehicles | D10.h Using Alcohol | 4 | D | Drunk driving |  |  | Motorcycle when drunk /Boating when drunk / Drunk driving as driver / Drunk driving -- friend / Drunk driving -- friend |  |  |  | Drinking and driving over the last year | I drive after drinking. |  |
| D4.h Driving motorized vehicles |  | 1 | D |  |  |  | Driving motorcycle / Motorcycle on icy road / Passenger on motorcycle |  |  |  |  |  |  |
| D5.a Sustenance ingesting |  | 4 | S |  |  | Avoiding high cholesterol foods |  | Avoid high cholesterol foods |  | Avoid foods high in cholesterol | Conscious effort to avoid fat and **cholesterol** |  |  |
| D5.a Sustenance ingesting |  | 7 | S | Taking vitamin supplements. |  | Taking vitamin supplements |  | Take vitamin supplements | Vitamins | Take daily vitamin supplements |  | I take vitamins | Taking vitamins / nutritional supplements |
| D5.a Sustenance ingesting |  | 4 | S |  |  | Minimizing sugar intake |  | Minimize sugar intake |  | Minimize sugar intake |  | I limit my intake of foods like coffee, sugar, fats, etc. |  |
| D5.a Sustenance ingesting |  | 1 | D |  |  |  |  |  |  |  |  | I take health food supplements (e.g., protein additives, wheat germ, bran, lecithin |  |
| D5.a.1 Eating |  | 6 | D |  | Breakfast every day | Eating breakfast every morning |  | Eat breakfast every morning |  | Eat breakfast every morning | Breakfast / Red meat consumption / Number of meals |  | Eating breakfast and/or regular meals / Eating bitter chocolate /Eating fresh and healthy food |
| D5.a.1.1 Eating Fruits |  | 3 | D | Eating five **fruit** and vegetables a day |  |  |  |  |  |  | Fruit consumption |  | Consuming fruits and vegetables |
| D5.a.1.2 Eating Vegetables |  | 2 | D | Eating five fruit and **vegetables** a day |  |  |  |  |  |  |  |  | Consuming fruits and **vegetables** |
| D5.a.1.3 Eating Fast food |  | 1 | D |  |  |  |  |  |  |  |  |  | Reducing consumption of foods that damage health (junk food, etc.) |
| D5.a.1.4 Eating Snack |  | 2 | D |  | No between-meal snacks |  |  |  |  |  | Number of snacks per day |  |  |
| D5.a.2 Drinking |  | 1 | S |  |  |  |  |  |  |  |  |  | Drinking plenty of water |
| D5.a.2.2 Drinking Coffee |  | 6 | D |  |  | Limiting daily caffeine intake |  | Limit daily caffeine intake |  | Limit daily caffeine intake /Drink coffee moderately or not at all | Coffee consumption | I limit my intake of foods like coffee, sugar, fats, etc. | Reducing coffee consumption |
| D5.a.4 Ingesting Fiber |  | 2 | D |  |  |  |  |  |  |  | Conscious effort to eat fiber |  | Eating fibers |
| D5.a.7 Ingesting Fat |  | 5 | D | Eating low fat diet |  | Avoiding foods high in saturated fats |  | Avoid saturated fat foods |  | Avoid foods high in saturated fats |  | I limit my intake of foods like coffee, sugar, fats, etc. |  |
| D5.a.9 Ingesting Sodium |  | 4 | D |  |  | Decreasing salt consumption |  | Decrease salt consumption |  | Decrease salt consumption | Adding salt to meals |  |  |
| D5.c Engaging in behavior related to maintaining one's health |  | 2 | S |  |  |  |  |  |  |  |  | I learn first aid techniques | Acquiring first aid knowledge |
| D5.c Engaging in behavior related to maintaining one's health |  | 2 | S |  |  |  |  |  |  |  |  | I discuss health with friends, neighbors, and relatives | Increasing knowledge in health issues |
| D5.c.1 Using health care/ participating in treatment |  | 1 | S |  |  |  |  |  |  |  |  |  | Undergo alternative medical treatments (**acupuncture**, etc.) |
| D5.c.1.2 Participating in Physician treatment |  | 4 | D | Visit doctor for health problem |  |  |  |  |  |  | Regular access to doctor | I see a doctor for regular checkups | Getting medical attention when necessary / Avoid doctors |
| D5.c.1.3 Participating in Physical therapy |  | 1 | D |  |  |  |  |  | Massages |  |  |  |  |
| D5.c.1.4 Participating in Dental Care |  | 4 | D | Visiting dentist yearly |  |  |  |  |  |  | Regular dental check-ups | I see a dentist for regular checkups | Regularly visiting a dental hygienist |
| D5.c.2 Taking medication |  | 4 | D |  |  | Taking OTC drugs exactly as directed |  |  | Herbal treatment |  |  | I avoid over-the-counter medicines | Keeping medication consumption to a minimum |
| D5.c.3 Adhering to medication/ treatment regime |  | 4 | D | Adhering to all prescribed medication |  | Taking prescriptions exactly as prescribed |  |  | Medication |  |  |  | Adhering to medical treatment (keeping to medication times, etc.) |
| D5.c.4 Doing a clinical screening |  | 6 | D | Attending health screening appointments if invited |  | An annual physical examination |  | Annual physical examination | Medical check-ups | Have an annual physical exam |  |  | Undergo periodic medical examinations |
| D5.c.4 Doing a clinical screening |  | 3 | S |  |  | Having a baseline exercise test before starting an exercise program |  | Baseline exercise test |  | Have a baseline exercise test |  |  |  |
| D5.c.4 Doing a clinical screening |  | 2 | S |  |  | Avoidance of unnecessary x-rays |  | Avoid unnecessary x-rays |  |  |  |  |  |
| D5.c.4 Doing a clinical screening |  | 2 | S |  |  |  |  | Annual exercise test |  | Have an annual exercise test |  |  |  |
| D5.c.4 Doing a clinical screening |  | 1 | S |  |  |  |  |  |  |  |  | I watch for possible signs of major health problems (e.g., cancer, hypertension, heart disease) |  |
| D5.c.4.1 Participating in testing |  | 1 | S |  |  |  |  |  |  |  | Blood pressure measurement |  |  |
| D5.c.4.1 Participating in testing |  | 1 | S |  |  |  |  |  |  |  | Cervical smear |  |  |
| D5.c.4.1 Participating in testing |  | 1 | S |  |  |  |  |  |  |  | Breast examination by health professional |  |  |
| D5.c.4.2 Self-testing |  | 2 | S | Performing self examination (**breasts** if female, testicular if male) |  |  |  |  |  |  | Breast self-examination |  |  |
| D5.c.4.2 Self-testing |  | 2 | S | Performing self examination (breasts if female, **testicular** if male) |  |  |  |  |  |  | Testicle self-examination |  |  |
| D5.c.5 Taking protective actions |  | 3 | S |  |  |  |  |  | Vaccinations |  |  | I get shots to prevent illness | Vaccine compliance |
| D5.c.5 Taking protective actions |  | 2 | S |  |  |  |  |  |  |  |  | I stay away from places where I might be exposed to germs | Avoiding being around sick people |
| D5.c.5 Taking protective actions |  | 2 | S |  |  | Wearing protective equipment/clothing around harmful substances |  | Protective equipment/clothing around harmful substances |  |  |  |  |  |
| D5.c.5 Taking protective actions |  | 2 | S |  |  |  |  |  |  |  |  | I keep emergency numbers near the phone | Preparing an emergency phone list |
| D5.c.5 Taking protective actions |  | 1 | S |  |  |  |  |  |  |  |  | I avoid areas of high pollution |  |
| D5.c.5 Taking protective actions |  | 1 | S |  |  |  |  |  |  |  |  |  | Installing emergency-call buttons in the home |
| D5.c.5 Taking protective actions |  | 1 | S |  |  |  |  |  |  |  |  |  | Keeping the environment clean and **avoiding polluted places** |
| D5.c.5 Taking protective actions |  | 1 | S |  |  |  |  |  |  |  |  | I destroy old and unused medicines |  |
| D5.c.5 Taking protective actions |  | 1 | S |  |  |  |  |  |  |  |  | I have a first aid kit in my home |  |
| D5.c.5 Taking protective actions |  | 1 | S |  |  |  |  |  | Sharing needles |  |  |  |  |
| D5.c.5 Taking protective actions |  | 1 | S |  |  |  | Reflector, walking in dark |  |  |  |  |  |  |
| D5.c.5 Taking protective actions |  | 1 | S |  |  |  |  |  |  |  |  | I avoid getting chilled |  |
| D5.c.5.1 Using sun protection |  | 2 | D | Using sunscreen of at least 15SPF |  |  |  |  |  |  |  |  | Protecting oneself from sun damage (using sun block, exposure times, etc.) |
| D5.c.5.3 Using safety restraints |  | 8 | D | Wearing a helmet when riding a bicycle |  | Always using seat belts | Seatbelt as passenger in front / Seatbelt as driver / Seatbelt as driver / Seatbelt as driver | Always use seat belts |  | Use a seat belt | Seat belt use | I wear a seat belt when in a car | Using road safety measures (seat belt, helmet, etc.) |
| D5.c.5.3 Using safety restraints |  | 1 | S |  |  |  | Life jacket in boats |  |  |  |  |  |  |
| D5.c.5.4 Using contraceptive devices |  | 1 | D |  |  |  |  |  |  |  |  |  | Having safe sex / using contraception |
| D5.c.5.4.1 Using condom |  | 2 | D |  |  | Use of condoms if one has multiple sexual partners |  |  | Sex without condom |  |  |  |  |
| D5.c.6 Managing diet and fitness |  | 4 | D |  |  | Eating a balanced diet |  | Eat a balanced diet |  | Eat a balanced diet |  | I eat a balanced diet |  |
| D5.c.6 Managing diet and fitness |  | 4 | D |  |  | Avoiding excessive caloric intake |  | Avoid excessive caloric intake |  | Avoid excess calories | Dieting |  |  |
| D5.c.6 Managing diet and fitness |  | 3 | D |  | Maintenance of approximate prescribed weight |  |  |  |  |  |  | I watch my weight | Engage in activities to maintain a proper bodyweight (BMI) |
| D5.c.6 Managing diet and fitness |  | 1 | S |  |  |  |  |  |  |  |  |  | Eating a diverse diet |
| D5.c.7 Recuperating |  | 1 | S |  |  |  |  |  |  |  |  |  | Going on vacation |
| D5.c.7 Recuperating |  | 1 | S |  |  |  |  |  |  |  |  |  | Outdoor hiking and **being in nature** |
| D5.c.7 Recuperating |  | 1 | S |  |  |  |  |  | Mud baths |  |  |  |  |
| D5.c.7.1 Sleeping |  | 7 | D |  | Sleep | Getting 7 hours of sleep each night |  | Get 7 hours sleep each night |  | Get 7 hours of sleep each night | Sleep time in hours | I get enough sleep | Sleeping enough and at regular hours / Sleeping in comfortable conditions (appropriate mattress and pillow etc.) |
| D5.c.7.2 Resting |  | 1 | D |  |  |  |  |  |  |  |  |  | Taking time to rest |
| D5.c.7.3 Doing relaxation exercises |  | 1 | S |  |  |  |  |  |  |  |  |  | Engaging in Oriental arts (yoga, **meditation**, etc.) |
| D5.c.7.3 Doing relaxation exercises |  | 3 | D |  |  | The practice of relaxation techniques |  | Practice relaxation methods |  | Regularly practice relaxation methods |  |  |  |
| D5.d Washing oneself |  | 1 | D |  |  |  |  |  |  |  |  |  | Keeping physical hygiene (showering, etc.) |
| D5.e.1 Flossing |  | 3 | D | Flossing teeth daily |  |  |  |  |  |  |  | I use dental floss regularly | Using dental floss regularly |
| D5.e.2 Toothbrushing |  | 4 | D | Brushing teeth |  |  |  |  |  |  | Tooth brushing | I brush my teeth regularly | Brushing teeth regularly |
| D6.b.1 Cleaning |  | 1 | D |  |  |  |  |  |  |  |  |  | **Keeping the environment clean** and avoiding polluted places |
| D6.c.2 Doing repairs |  | 2 | D |  |  |  |  |  |  |  |  | I fix broken things around my home right away / I check the condition of electrical appliances, the car, etc.to avoid accidents | Organizing the environment to prevent accidents |
| D6.Engaging in domestic life activities |  | 1 | S |  |  |  |  |  |  |  |  |  | Spending quality time with one’s partner |
| D7. Engaging in interpersonal interactions and relationships |  | 1 | S |  |  |  |  |  |  |  |  |  | Having good relationships with one’s family |
| D7. Engaging in interpersonal interactions and relationships |  | 1 | D |  |  |  |  |  |  |  |  |  | Communicating well with people / having good human relations |
| D7.a.1.2 Hiring a prostitute |  | 1 | D |  |  |  |  |  | Sex with prostitute |  |  |  |  |
| D7.a.2.2 Having intercourse |  | 1 | D |  |  |  |  |  |  |  |  |  | Having fulfilling sexual intercourse |
| D8.c Engaging in behaviors related to acquiring, keeping and terminating a job |  | 1 | D |  |  |  |  |  |  |  |  |  | Finding a job you like / pleasant working environment |
| D8.e Using technology |  | 1 | D |  |  |  |  |  |  |  |  |  | Avoiding talking on a mobile phone or using a hands-free phone |
| D9.a.1 Engaging in behavior related to spirituality |  | 1 | D |  |  |  |  |  |  |  |  | I pray or live by principles of religion |  |
| D9.b.1 Participating in hobbies/activities |  | 1 | S |  |  |  | Diving from 5 meters |  |  |  |  |  |  |
| D9.b.1 Participating in hobbies/activities |  | 1 | D |  |  |  |  |  |  |  |  | I engage in activities or hobbies where accidents are possible (e.g., motorcycle riding, skiing, using power tools, sky or skin diving, hang gliding, etc.) |  |
| D9.b.1 Participating in hobbies/activities |  | 2 | D |  |  |  | Skiing -- steep mountain |  |  |  |  | I engage in activities or hobbies where accidents are possible (e.g., motorcycle riding, **skiing**, using power tools, sky or skin diving, hang gliding, etc.) |  |
| D9.b.1.2 Playing sports |  | 1 | D |  |  |  | Risky sports |  |  |  |  |  |  |
| D9.b.1.2 Playing sports |  | 1 | D |  |  |  |  |  |  |  |  | I choose my spare time activities to help me relax |  |
| D9.c.1.1.1 Volunteering |  | 1 | D |  |  |  |  |  |  |  |  |  | Volunteering and helping others |
| D9.c.1.1.5 Donating blood |  | 1 | D |  |  |  |  |  | Giving blood |  |  |  |  |
| D9.d Engaging in prosocial behavior |  | 1 | D |  |  |  |  |  |  |  |  |  | Creating and maintaining supportive social relationships |
| D9.e.1 Engaging in non-aggressive antisocial behavior |  | 1 | S |  |  |  |  |  |  |  |  | I cross busy streets in the middle of the block / I take chances when crossing the street |  |
| D9.e.1 Engaging in non-aggressive antisocial behavior |  | 1 | S |  |  |  |  |  |  |  |  | I carefully obey traffic rules so I don't have accidents |  |
| D9.e.1 Engaging in non-aggressive antisocial behavior |  | 1 | S |  |  |  |  |  |  |  |  | I cross the street against the stop light |  |
| D9.e.1.14 Speeding |  | 4 | D | Exceeding posted speed limit on public highway |  |  | Speeding, at 90 km-h / Breaking speed limit in gen. / Speeding, at 120 km-h |  |  |  | Driving within speed limit | I speed while driving |  |
| D10.a Quitting | D10.g Using Tobacco | 2 | S |  |  | Elimination of cigarette, **pipe** or cigar smoking |  | Eliminate pipe smoking |  |  |  |  |  |
| D10.b Abstaining | D10.h Using Alcohol | 8 | D | Binge drinking / Daily drinking of alcohol (exceeding recommended limit) / Daily drinking of alcohol (exceeding recommended limit) | Moderate or no use of alcohol | Drinking alcohol in moderation / avodiance of alcohol altogether |  | Drink alcohol moderately / Drink no alcohol at all |  | Drink alcohol moderately or not at all | Alcohol consumption | I do not drink alcohol | Avoiding excessive alcohol consumption |
| D10.b Abstaining | D10. Engaging in mood / state changing activities and behavior | 3 | D | Using illegal drugs |  |  |  |  |  |  |  | I don't take chemical substances which might injure my health (e.g., food additives, drugs, stimulants) | Avoid using drugs |
| D10.g.2 Smoking Cigar(ette) | D10.b Abstaining | 9 | D | Smoking | No smoking | Elimination of **cigarette**, pipe or **cigar** smoking |  | Eliminate **cigarette** smoking / Eliminate **cigar** smoking | Smoking | Eliminate cigarette smoking | Cigarette smoking | I don't smoke | Avoiding smoking |
| D10.j Using Opiates |  | 1 | D |  |  |  |  |  | Heroin |  |  |  |  |
| D10.m Using Marijuana |  | 1 | D |  |  |  |  |  | Weed |  |  |  |  |

^1^D=Directly fits in IC-Behavior/ S=Sub-fits as a direct descendant of a node in IC-Behavior / N=Not a fit with IC-Behavior.

**References**

1. Nudelman G, Shiloh S. Mapping health behaviors: Constructing and validating a common-sense taxonomy of health behaviors. Social Science & Medicine. 2015;146:1-10.

2. Vickery CE, Cotugna N. Dietitians' beliefs about the importance of health promotion practices. Journal of community health. 1990;15(5):319-27.

3. Sobal J, Valente CM, Muncie Jr HL, Levine DM, Deforge BR. Physicians' beliefs about the importance of 25 health promoting behaviors. American journal of public health. 1985;75(12):1427-8.

4. Harvey RD, Afful SE. Racial typicality, racial identity, and health behaviors: a case for culturally sensitive health interventions. Journal of Black Psychology. 2011;37(2):164-84.

5. Gorin SS. Student nurse opinions about the importance of health promotion practices. Journal of community health. 1992;17(6):367-75.

6. Vickers RR, Conway TL, Hervig LK. Demonstration of replicable dimensions of health behaviors. Preventive medicine. 1990;19(4):377-401.

7. Williams B, Onsman A, Brown T. Exploratory factor analysis: A five-step guide for novices. Australasian Journal of Paramedicine. 2010;8(3).

8. McEachan RR, Lawton RJ, Conner M. Classifying health‐related behaviours: Exploring similarities and differences amongst behaviours. British Journal of Health Psychology. 2010;15(2):347-66.

9. Matarazzo JD. Behavioral immunogens and pathogens: Psychology's newest challenge. Professional Psychology: Research and Practice. 1983;14(3):414.

10. Røysamb E, Rise J, Kraft P. On the structure and dimensionality of health-related behaviour in adolescents. Psychology and Health. 1997;12(4):437-52.

11. Wardle J, Steptoe A. The European Health and Behaviour Survey: rationale, methods and initial results from the United Kingdom. Social science & medicine. 1991;33(8):925-36.
