# Supplementary material for: IC-Behavior: An interdisciplinary taxonomy of behaviors
Source: PLoS One. 2021 Sep 17;16(9):e0252003. doi: 10.1371/journal.pone.0252003 (PMC8448352; doi:10.1371/journal.pone.0252003)
Supplement: S4 File — (DOCX) [file pone.0252003.s004.docx]

**S4 File. IC-Behavior Taxonomy with Definitions, and Thesaurus Links**

| **L1** | **L2** | **L3** | **L4** | **L5** | **L6** | **Definition** | **Reference** | **NCI Thesaurus Code** | **NCI Metathesaurus Code** |
| --- | --- | --- | --- | --- | --- | --- | --- | --- | --- |
| D. Engaging in activities / participating [ICF: D] |  | | | | | The condition in which things are happening or being done and/or the action of taking part in something. | Oxford Dictionary | C25608 | C0679823 |
|  | D1. Engaging in learning and applying knowledge [ICF: D1] |  | | | | The acquisition or application of knowledge or skills through study, experience, or being taught. | Oxford Dictionary | C19369 | C0023185 |
|  |  | D1.a Watching [ICF: D110] |  |  |  | "The action of observing attentively, typically for a period of time." | Google dictionary (adapted) |  |  |
|  |  | D1.a Watching [ICF: D110] | D1.a.1 Watching TV |  |  | The act of watching television. | NCI Thesaurus | C106685 | C3827501 |
|  |  | D1.b Listening [ICF: D115] |  |  |  | To pay attention to someone or something in order to hear what is being said, sung, played, etc. | Merriam-Webster |  | C2584303 |
|  |  | D1.b Listening [ICF: D115] | D1.b.1 Listening to music |  |  | To pay attention to in order to hear to music that is being sung / played. | Merriam-Webster (adapted) |  | C2136029 |
|  |  | D1.c Reading [ICF: D166] |  |  |  | "The action of looking at and comprehending the meaning of (written or printed matter) by mentally interpreting the characters or symbols of which it is composed." | Google dictionary | C48222 | C0034754 |
|  |  | D1.c Reading [ICF: D166] | D1.c.1 Reading aloud |  |  | The action of looking at and comprehending the meaning of (written or printed matter) by mentally interpreting the characters or symbols of which it is composed and simultaneously verbalizing the read content. | Google dictionary (adapted) |  |  |
|  |  | D1.d Writing [ICF: D170] | D1.c.1 Reading aloud | D1.c.1.1 Reading aloud for child |  | The action of looking at and comprehending the meaning of (written or printed matter) by mentally interpreting the characters or symbols of which it is composed and simultaneously verbalizing the read content to a child. | Google dictionary (adapted) |  |  |
|  |  | D1.e Drawing |  |  |  | "The activity of composing text" | Oxford Dictionary (adapted) | C73994 | CL448932 |
|  |  | D1.a Drawing |  |  |  | The act or art of making a picture, image, etc., with a pencil, pen, marker, chalk, etc., but usually not with paint. | Merriam-Webster | C54353 | CL448807 |

|  | D3. Communicating [ICF: D3] |  |  |  |  | The act or process of using words, sounds, signs, or behaviors to express or exchange information or to express your ideas, thoughts, feelings, etc., to someone else. | Merriam-Webster | C16452 | C0009452 |
| --- | --- | --- | --- | --- | --- | --- | --- | --- | --- |
|  |  | D3.a Receiving communications [ICF: D310-D329] |  |  |  | The act or process of receiving words, sounds, signs, or behaviors that express information. | Merriam-Webster (adapted) |  |  |
|  |  | D3.a Receiving communications [ICF: D310-D329] | D3.a1 Listening to spoken messages |  |  | "The action of receiving information or another one’s feelings in speech" | Oxford Dictionary |  |  |
|  |  | D3.a Receiving communications [ICF: D310-D329] | D3.a2 Gathering information |  |  | "The act of collecting information." | Collins dictionary |  |  |
|  |  | D3.a Receiving communications [ICF: D310-D329] | D3.a3 Looking |  |  | Direct one’s gaze in a specified direction. | Oxford Dictionary |  |  |
|  |  | D3.a Receiving communications [ICF: D310-D329] | D3.a3 Looking | D3.a3.1 Looking at erotic material |  | Direct one’s gaze at erotic material. | Oxford Dictionary (adapted) |  |  |
|  |  | D3.a Receiving communications [ICF: D310-D329] |  |  |  | The act or process of generating words, sounds, signs, or behaviors to express information or to express your ideas, thoughts, feelings, etc., to someone else. | Merriam-Webster (adapted) |  |  |
|  |  | D3.b Producing communications [ICF: D330-D349] | D3.b.1 Producing verbal communications |  |  | The act or process of generating words to express information or to express your ideas, thoughts, feelings, etc., to someone else. | Merriam-Webster (adapted) |  |  |
|  |  | D3.b Producing communications [ICF: D330-D349] | D3.b.1 Producing verbal communications | D3.b.1.1 Speaking |  | "The action of expressing one's thoughts and feelings in spoken language (includes, for example, whining)" | Google dictionary | C86914 | C0234856 |
|  |  | D3.b Producing communications [ICF: D330-D349] | D3.b.1 Producing verbal communications | D3.b.1.1 Speaking | D3.b1.1 Speaking to a child | "The [parent] talks to the child." | [1] |  |  |
|  |  | D3.b Producing communications [ICF: D330-D349] | D3.b.1 Producing verbal communications | D3.b1.2 Writing messages |  | "The action of communication through a sequence of letters, words, or symbols marked on a surface" | Oxford Dictionary (adapted) |  |  |
|  |  | D3.b Producing communications [ICF: D330-D349] | D3.b.2 Producing non-verbal communications |  |  | The act of communication not involving or using words. | Merriam-Webster (adapted) | C1285674 | C1285674 |
|  |  | D3.b Producing communications [ICF: D330-D349] | D3.b.2 Producing non-verbal communications | D3.b.2.1 Producing facial expressions |  | The look on someone's face (such as smiling, frowning, etc). | Merriam-Webster (adapted) |  | C0015457 |
|  |  | D3.b Producing communications [ICF: D330-D349] | D3.b.2 Producing non-verbal communications | D3.b.2.2 Laughing |  | Make the spontaneous sounds and movements of the face and body that are the instinctive expressions of lively amusement and sometimes also of derision. | Oxford Dictionary |  |  |
|  |  | D3.b Producing communications [ICF: D330-D349] | D3.b.2 Producing non-verbal communications | D3.b.2.3 Crying |  | Producing tears often while making loud sounds because of pain, sorrow, or other strong emotions. | Merriam-Webster (adapted) | C77963 | C0010399 |
|  |  | D3.b Producing communications [ICF: D330-D349] | D3.b.2 Producing non-verbal communications | D3.b.2.4 Touching |  | "Bring one’s hand or another part of one’s body into contact with another person or object" | Oxford Dictionary (adapted) |  |  |
|  |  | D3.b Producing communications [ICF: D330-D349] | D3.b.2 Producing non-verbal communications | D3.b.2.5 Producing body gestures/postures | | Producing a movement of your body that shows or emphasizes an idea or a feeling. | Merriam-Webster (adapted) |  | C0017510 |
|  |  | D3.b Producing communications [ICF: D330-D349] | D3.b.2 Producing non-verbal communications | D3.b.2.6 Producing drawings or photographs |  | "Making picture(s) or diagram(s) with a pencil, pen, or crayon rather than paint; taking photographs." | Oxford Dictionary (adapted) |  |  |
|  |  | D3.c Engaging in interactive communication (both receiving and producing) |  |  |  | Exchange of information by the use of words, sounds or signs between two or more people. | Merriam-Webster (adapted) | C16452 | C0009452 |
|  | D4. Moving/exercising [ICF: D4] |  |  |  |  | To change position or make one´s body change position in a way that can be seen, heard or felt; physical activity that someone does to stay healthy or become stronger. | Merriam-Webster (adapted) | C121370 | CL493908 |
|  |  | D4.a Stretching |  |  |  | "Straighten or extend one’s body or a part of one’s body to its full length, typically so as to tighten one’s muscles." | Oxford dictionary |  |  |
|  |  | D4.b Crawling [ICF:d4550] |  |  |  | Move forward on the hands and knees or by dragging the body close to the ground. | Oxford dictionary | C1635178 | C1635178 |
|  |  | D4.c Walking |  |  |  | Move at a regular pace by lifting and setting down each foot in turn, never having both feet off the ground at once. | Merriam-Webster | C73554 | C0080331 |
|  |  | D4.d Running |  |  |  | To move using the legs, going faster than when walking. | Merriam-Webster (adapted) | C16567 | C0015259 |
|  |  | D4.e Biking |  |  |  | "The act of riding a bicycle." | Merriam-Webster Dictionary | C0005377 | C0005377 |
|  |  | D4.f Swimming |  |  |  | The sport or activity of moving through water by moving your arms and legs. | Merriam-Webster Dictionary | C94738 | C0039003 |
|  |  | D4.g Aerobics |  |  |  | A system of exercises often done by a group of people while music is playing | Merriam-Webster Dictionary | C126241 | C0005377 |
|  |  | D4.h Driving motorized vehicles |  |  |  | The act of control and operation of a motorized vehicle. | Oxford Dictionary (adapted) | C95548 | C0004379 |
|  | D5. Engaging in self-care behavior [ICF: D5] |  |  |  |  | The provision of what is necessary for one´s health, welfare, maintenance, and protection. | Oxford Dictionary |  |  |
|  |  | D5.d Washing oneself |  |  |  | Clean oneself, commonly with soap and water. | Oxford Dictionary | C65077 | C0441648 |
|  |  | D5.e Caring for body parts |  |  |  | The act of caring for body parts in order to ensure what is needed for their health or protection. | Oxford Dictionary (adapted) |  |  |
|  |  | D5.e Caring for body parts | D5.e.1 Flossing |  |  | "The use of waxed or unwaxed synthetic thread to dislodge plaque, foods and microbes from the lateral borders of teeth." | Segen's Medical Dictionary | C63735 | C1880794 |
|  |  | D5.e Caring for body parts | D5.e.1 Toothbrushing |  |  | "The action of using a toothbrush to clean the teeth." | Merriam-Webster Dictionary | C63734 | C1880794 |
|  |  | D5.a Sustenance ingesting |  |  |  | Nourishment. | Oxford Dictionary | C0232478 | C0232478 |
|  |  | D5.a Sustenance ingesting | D5.a.1 Eating [ICF: D550] |  |  | Put (food) into the mouth and chew and swallow it. | Oxford Dictionary | C86069 | C0013470 |
|  |  | D5.a Sustenance ingesting | D5.a.1 Eating [ICF: D550] | D5.a.1.1 Eating Fruits |  | "The consumption of the usually edible reproductive body of a seed plant." | Merriam-Webster dictionary | C71972 | C0016767 |
|  |  | D5.a Sustenance ingesting | D5.a.1 Eating [ICF: D550] | D5.a.1.2 Eating Vegetables |  | The consumption of the edible part of a usually herbaceous plant (such as the cabbage, bean, or potato). | Merriam-Webster dictionary |  |  |
|  |  | D5.a Sustenance ingesting | D5.a.1 Eating [ICF: D550] | D5.a.1.3 Eating Fast food |  | "The consumption of easily prepared food served in restaurants and snack bars as a quick meal or to be taken out." | Oxford dictionary |  |  |
|  |  | D5.a Sustenance ingesting | D5.a.1 Eating [ICF: D550] | D5.a.1.4 Eating Snack |  | "The consumption of a small amount of food between meals." | Google dictionary |  |  |
|  |  | D5.a Sustenance ingesting | D5.a.1 Eating [ICF: D550] | D5.a.1.4 Eating Snack | D5.a.1.4.1 Eating Candy | "The consumption of a sweet food made with sugar or syrup combined with fruit, chocolate, or nuts." | Google dictionary |  |  |
|  |  | D5.a Sustenance ingesting | D5.a.1 Eating [ICF: D550] | D5.a.1.4 Eating Snack | D5.a.1.4.2 Eating Potato chips | "The consumption of a wafer-thin slice of potato fried or baked until crisp." | Google dictionary |  |  |
|  |  | D5.a Sustenance ingesting | D5.a.1 Eating [ICF: D550] | D5.a.1.5 Eating Ice cream |  | "The consumption of a sweet flavored frozen food containing cream or butterfat and usually eggs." | Merriam-Webster dictionary |  |  |
|  |  | D5.a Sustenance ingesting | D5.a.1 Eating [ICF: D550] | D5.a.1.6 Eating Cereal |  | "The consumption of a plant (as a grass) yielding starchy grain suitable for food." | Merriam-Webster dictionary |  |  |
|  |  | D5.a Sustenance ingesting | D5.a.1 Eating [ICF: D550] | D5.a.1.7 Eating Baked goods |  | "Consumption of food products that have been baked in an oven." | Cambridge Dictionary |  |  |
|  |  | D5.a Sustenance ingesting | D5.a.2 Drinking [ICF: D560] |  |  | To take a liquid into your mouth and swallow it. | Merriam-Webster |  |  |
|  |  | D5.a Sustenance ingesting | D5.a.2 Drinking [ICF: D560] | D5.a.2.1 Drinking Soda |  | "The consumption of a sweet, carbonate, nonalcoholic beverage." | Merriam-Webster dictionary |  |  |
|  |  | D5.a Sustenance ingesting | D5.a.2 Drinking [ICF: D560] | D5.a.2.2 Drinking Coffee |  | "The consumption of a drink made from the roasted and ground beanlike seeds of a tropical shrub." | Google Dictionary |  |  |
|  |  | D5.a Sustenance ingesting | D5.a.2 Drinking [ICF: D560] | D5.a.2.3 Drinking Smoothie |  | "Consumption of a thick, smooth drink of fresh fruit pureed with milk, yogurt, or ice cream." | Google Dictionary |  |  |
|  |  | D5.a Sustenance ingesting | D5.a.2 Drinking [ICF: D560] | D5.a.2.4 Drinking Milk |  | Consumption of an opaque white fluid rich in fat and protein, secreted by female mammals for the nourishment of their young:" | Oxford Dictionary |  |  |
|  |  | D5.a Sustenance ingesting | D5.a.3 Ingesting Energy |  |  | "The strength and vitality provided by food or drink consumed for sustaining physical or mental activity" | Oxford Dictionary (adapted) |  |  |
|  |  | D5.a Sustenance ingesting | D5.a.4 Ingesting Fiber |  |  | Ingesting mostly indigestible material in food that stimulates the intestine to peristalsis — called also bulk, roughage. | Merriam-Webster (adapted) | C68492 | CUI C0012173 |
|  |  | D5.a Sustenance ingesting | D5.a.5 Ingesting Calcium |  |  | Ingesting a silver-white divalent metallic element of the alkaline-earth group occurring only in combination. | Merriam-Webster (adapted) | C68241 | CUI C0006726 |
|  |  | D5.a Sustenance ingesting | D5.a.6 Ingesting Protein |  |  | Ingesting a group of complex organic macromolecules composed of one or more chains (linear polymers) of alpha-L-amino acids linked by peptide bonds and ranging in size from a few thousand to over 1 million Daltons. Proteins are fundamental genetically encoded components of living cells with specific structures and functions dictated by amino acid sequence. | NCI Thesaurus (adapted) | C17021 | CUI C0033684 |
|  |  | D5.a Sustenance ingesting | D5.a.7 Ingesting Fat |  |  | Ingesting a class of food constituents, also known as fats and oils, that serve as a source of energy and essential fatty acids in animals. | NCI Thesaurus (adapted) | C2317 | CUI C0012171 |
|  |  | D5.a Sustenance ingesting | D5.a.8 Ingesting Sweetener |  |  | Ingesting a food additive which duplicates the effect of sugar on the taste, but with fewer calories. They are also called sugar substitutes. | NCI Thesaurus (adapted) | C283 | CUI C0003920 |
|  |  | D5.a Sustenance ingesting | D5.a.9 Ingesting Sodium |  |  | Ingesting a soft silver-white element that is found in salt, baking soda, and other compounds. | Merriam-Webster (adapted) | C68287 | CUI C0037570 |
|  |  | D5.a Sustenance ingesting | D5.a.10 Ingesting Carbohydrate |  |  | Ingesting any one of various substances found in certain foods (such as bread, rice, and potatoes) that provide your body with heat and energy and are made of carbon, hydrogen, and oxygen. | Merriam-Webster (adapted) | C68470 | C0012170 |
|  |  | D5.a Sustenance ingesting | D5.a11 Ingesting Sugar |  |  | Ingesting a white crystalline carbohydrate, typically sucrose, used as a sweetener and preservative. | NCI Thesaurus (adapted) | C71939 | CUI C0242209 |
|  |  | D5.a Sustenance ingesting | D5.a12 Ingesting Cholesterol |  |  | Ingesting an animal sterol found in the body tissues (and blood plasma) of vertebrates. | NCI Thesaurus (adapted) | C369 | CUI C0008377 |
|  |  | D5.a Sustenance ingesting | D5.a13 Ingesting Vitamins |  |  | Ingesting a group of organic food substances found in animals and plants in small quantities. They must be supplied in diet or dietary supplement and are essential for normal metabolism. | NCI Thesaurus (adapted) | C944 | C0042890 |
|  |  | D5.b Help-seeking |  |  |  | Request assistance to make it easier for someone to do a job, to deal with a problem, etc. | Merriam-Webster (adapted) | C0683301 | C0683301 |
|  |  | D5.c Engaging in behavior related to maintaining one's health [ICF: D5702] |  |  |  | Activities undertaken for the purpose of preventing or detecting disease or for improving health and well being. | [2, p. 2] |  |  |
|  |  | D5.c Engaging in behavior related to maintaining one's health [ICF: D5702] | D5.c.1 Using health care/ participating in treatment |  |  | The actions of prevention, treatment, and management of illness and the preservation of mental and physical well-being through the services offered by the medical and allied health professions. | NCI Metathesaurus | C16205 | CL414488 |
|  |  | D5.c Engaging in behavior related to maintaining one's health [ICF: D5702] | D5.c.1 Using health care/ participating in treatment | D5.c.1.1 Participating in Psychological treatment |  | The act of receiving professional psychological or emotional counseling. | [3] | C0841584 | C0841584 |
|  |  | D5.c Engaging in behavior related to maintaining one's health [ICF: D5702] | D5.c.1 Using health care/ participating in treatment | D5.c.1.2 Participating in Physician treatment |  | The actions of treatment of illness through the services offered by the medical professions. | NCI Metathesaurus (adapted) |  |  |
|  |  | D5.c Engaging in behavior related to maintaining one's health [ICF: D5702] | D5.c.1 Using health care/ participating in treatment | D5.c.1.3 Participating in Physical therapy |  | "The act of recieving treatment for disease, injury, or deformity by physical methods such as massage, heat treatment, and exercise rather than by drugs or surgery." | Merriam-Webster Dictionary | C0949766 | C0949766 |
|  |  | D5.c Engaging in behavior related to maintaining one's health [ICF: D5702] | D5.c.1 Using health care/ participating in treatment | D5.c.1.4 Participating in Dental Care |  | The act of seeking care for the functional and esthetic aspects of the hard and soft tissues of the oral and maxillofacial regions. | American Dental Association House of Delegates | C0011331 | C0011331 |
|  |  | D5.c Engaging in behavior related to maintaining one's health [ICF: D5702] | D5.c.1 Using health care/ participating in treatment | D5.c.1.5 Participating in Group support/treatment |  | The act of attending group support sessions led by a trained professional in order to attain healing or recovery. | Center for Substance Abuse Treatment | C1883225 | C1883225 |
|  |  | D5.c Engaging in behavior related to maintaining one's health [ICF: D5702] | D5.c.2 Taking medication |  |  | The act of taking a dose of medication into one's body (e.g., by swallowing, drinking, or inhaling). | Merriam-Webster (adapted) |  |  |
|  |  | D5.c Engaging in behavior related to maintaining one's health [ICF: D5702] | D5.c.3 Adhering to medication/ treatment regime |  |  | The act of abiding by a stated treatment plan or protocol. | NCI Thesaurus | C25729 | C1510802 |
|  |  | D5.c Engaging in behavior related to maintaining one's health [ICF: D5702] | D5.c.4 Doing a clinical screening |  |  | The act of participating in a systematic clinical examination or assessment. | NCI Metathesaurus (adapted) | C48261 | C1710032 |
|  |  | D5.c Engaging in behavior related to maintaining one's health [ICF: D5702] | D5.c.4 Doing a clinical screening | D5.c.4.1 Participating in testing |  | The act of participating in a diagnostic procedure for critical evaluation of the presence or absence of a condition, disease or change in function. | NCI Thesaurus (adapted) | C15336 | C1710032 |
|  |  | D5.c Engaging in behavior related to maintaining one's health [ICF: D5702] | D5.c.4 Doing a clinical screening | D5.c.4.1 Participating in testing | D5.c.4.1.1 Participate in baseline exercise test | The act of establishing an exercise function starting point to which change may be compared. | NCI Thesaurus (adapted) | C25213 | C1442488 |
|  |  | D5.c Engaging in behavior related to maintaining one's health [ICF: D5702] | D5.c.4 Doing a clinical screening | D5.c4.2 Self-testing |  | The act of checking own body for symptoms of illness. | Google dictionary |  |  |
|  |  | D5.c Engaging in behavior related to maintaining one's health [ICF: D5702] | D5.c.5 Taking protective actions |  |  | To cover or shield from exposure, injury, damage, or destruction for purposes of maintaining one's health. | Merriam-Webster (adapted) |  |  |
|  |  | D5.c Engaging in behavior related to maintaining one's health [ICF: D5702] | D5.c.5 Taking protective actions | D5.c.5.1 Using sun protection |  | To cover or shield from sun exposure, injury, or damage, for purposes of maintaining one's health. | Merriam-Webster (adapted) |  |  |
|  |  | D5.c Engaging in behavior related to maintaining one's health [ICF: D5702] | D5.c.5 Taking protective actions | D5.c.5.2 Using hearing protection |  | The use of hearing protection devices when exposed to high noise | [4] |  |  |
|  |  | D5.c Engaging in behavior related to maintaining one's health [ICF: D5702] | D5.c.5 Taking protective actions | D5.c.5.3 Using safety restraints |  | The use of devices (seat belt, car seat, booster seat, life belt, safety harness, etc.) in order to prevent getting hurt. | WHO; free Dictionary (adapted) |  |  |
|  |  | D5.c Engaging in behavior related to maintaining one's health [ICF: D5702] | D5.c.5 Taking protective actions | D5.c.5.4 Using contraceptive devices |  | Use of a device or drug that serves to prevent a woman from becoming pregnant. | Merriam-Webster (adapted) | C42734 | C0009886 |
|  |  | D5.c Engaging in behavior related to maintaining one's health [ICF: D5702] | D5.c.5 Taking protective actions | D5.c.5.4 Using contraceptive devices | D5.c.5.4.1 Using condom | Use of a sheath commonly of rubber worn over the penis (as to prevent conception or venereal infection during coitus) or a device that is designed to be inserted into the vagina before coitus and that resembles in form and function the condom used by males. | Merriam-Webster (adapted) |  | C0679782 |
|  |  | D5.c Engaging in behavior related to maintaining one's health [ICF: D5702] | D5.c.5 Taking protective actions | D5.c5.5 Taking vaccinations |  | Receiving administration of vaccines to stimulate the host's immune response. This includes any preparation intended for active immunological prophylaxis or treatment. | NCI Thesaurus | C15346 | C0042196 |
|  |  | D5.c Engaging in behavior related to maintaining one's health [ICF: D5702] | D5.c.6 Managing diet and fitness [ICF: D5701] | |  | To eat and drink sparingly or according to prescribed rules and to exercise for the purpose of improving or maintaining health. | Merriam-Webster (adapted) |  |  |
|  |  | D5.c Engaging in behavior related to maintaining one's health [ICF: D5702] | D5.c.7 Recuperating |  |  | "Actions taken to recover from sickness or exhaustion and to regain health or strength." | Merriam-Webster Dictionary |  |  |
|  |  | D5.c Engaging in behavior related to maintaining one's health [ICF: D5702] | D5.c.7 Recuperating | D5.c.7.1 Sleeping |  | "Putting oneself into a condition of body and mind in which the nervous system is relatively inactive, the eyes closed, the postural muscles relaxed, and consciousness practically suspended." | Merriam-Webster Dictionary | C73425 | C0037313 |
|  |  | D5.c Engaging in behavior related to maintaining one's health [ICF: D5702] | D5.c.7 Recuperating | D5.c.7.2 Resting |  | "The act of ceasing work or movement in order to relax, refresh oneself, or recover strength." | Google Dictionary | C28185 | C0035253 |
|  |  | D5.c Engaging in behavior related to maintaining one's health [ICF: D5702] | D5.c.7 Recuperating | D5.c.7.3 Doing relaxation exercises |  | "The act of performing exercises to reduce stress, muscle tension, and anxiety in the body." | Google Dictionary |  | C0203993 |
|  | D6. Engaging in domestic life activities [ICF: D6] |  |  |  |  | "Carrying out domestic and everyday actions and tasks" | [5, p. 153]. |  |  |
|  |  | D6.a Preparing meals [ICF: D630] |  |  |  | "The practice or skill of preparing food by combining, mixing, and heating ingredients." | Google dictionary | C110951 | C3828485 |
|  |  | D6.b Doing housework [ICF: D640] |  |  |  | The act of engaging in housekeeping. | Google dictionary | C98072 | C1571726 |
|  |  | D6.b Doing housework [ICF: D640] | D6.b.1 Cleaning [ICF: D6401] |  |  | An act of making a place clean or tidy. | Google dictionary |  |  |
|  |  | D6.c Caring for household objects and non-human members [ICF: D650] |  |  |  | To keep something in the household safe and provided for. | Oxford dictionary (adapted) |  | C4035663 |
|  |  | D6.c Caring for household objects and non-human members [ICF: D650] | D6.c.1 Gardening |  |  | "The activity of tending and cultivating a garden." | Google dictionary |  | C0868963 |
|  |  | D6.c Caring for household objects and non-human members [ICF: D650] | D6.c.2 Doing repairs |  |  | To keep an animal in the household safe and provided for. | Oxford dictionary (adapted) |  | C2712952 |
|  |  | D6.c Caring for household objects and non-human members [ICF: D650] | D6.c.3 Taking care of animals |  |  | "To fix or mend something suffering from damage or a fault." | Google dictionary |  |  |
|  | D7. Engaging in interpersonal interactions and relationships [ICF: D7] |  |  |  |  | The act of engaging in short or long-term association between two or more people, including kinship relations, romantic, business, and social interactions. | NCI Thesaurus (adapted) | C92454 | C0021797 |
|  |  | D7.a Engaging in behaviors related to intimate relationships [ICF: D770] |  |  |  | Engaging in behaviors related to private and personal relationships; this may be in a sexual way. | Oxford dictionary (adapted) |  | C2584308 |
|  |  | D7.a Engaging in behaviors related to intimate relationships [ICF: D770] | D7.a.1 Engaging in behavior related to prostitution |  |  | Engaging in sexual activity with someone in return for payment or as a customer. | Oxford dictionary (adapted) |  | C0033595 |
|  |  | D7.a Engaging in behaviors related to intimate relationships [ICF: D770] | D7.a.1 Engaging in behavior related to prostitution | D7.a.1.1 Working as a prostitute |  | "The practice or occupation of engaging in sexual activity with someone for payment." | Google dictionary |  |  |
|  |  | D7.a Engaging in behaviors related to intimate relationships [ICF: D770] | D7.a.1 Engaging in behavior related to prostitution | D7.a.1.2 Hiring a prostitute |  | The act of employing a person to engage in sexual activity for payment. | Oxford dictionary (adapted) |  |  |
|  |  | D7.a Engaging in behaviors related to intimate relationships [ICF: D770] | D7.a.2 Engaging in sexually intimate actions |  |  | The act of private and personal sexual interactions. | Google dictionary | C17127 | C0036864 |
|  |  | D7.a Engaging in behaviors related to intimate relationships [ICF: D770] | D7.a.2 Engaging in sexually intimate actions | D7.a.2.1 Having non-intercourse sexual contact |  | The act of physical sexual contact such as petting, engaging in amorous embracing, caressing, and kissing between individuals, but without intercourse. | Merriam-Webster (adapted) |  |  |
|  |  | D7.a Engaging in behaviors related to intimate relationships [ICF: D770] | D7.a.2 Engaging in sexually intimate actions | D7.a.2.2 Having intercourse |  | The act of physical sexual contact between individuals that involves the genitalia of at least one person. | Merriam-Webster | C72881 | C0009253 |
|  |  | D7.a Engaging in behaviors related to intimate relationships [ICF: D770] | D7.a.2 Engaging in sexually intimate actions | D7.a.2.2 Having intercourse | D7.a.2.2.1 Having Oral intercourse | The act of sexual activity in which the genitals of one partner are stimulated by the mouth of the other; fellatio or cunnilingus. | Google dictionary |  | C0556632 |
|  |  | D7.a Engaging in behaviors related to intimate relationships [ICF: D770] | D7.a.2 Engaging in sexually intimate actions | D7.a.2.2 Having intercourse | D7.a.2.2.2 Having Vaginal intercourse | Sexual activity in which a person puts a natural or artificial penis into the vagina of a woman. | Merriam-Webster |  | C0556623 |
|  |  | D7.a Engaging in behaviors related to intimate relationships [ICF: D770] | D7.a.2 Engaging in sexually intimate actions | D7.a.2.2 Having intercourse | D7.a.2.2.3 Having Anal intercourse | Sexual activity in which a person puts a natural or artificial penis into the anus of another. | Merriam-Webster |  | C0556628 |
|  | D8. Engaging in behavior related to major life areas [ICF: D8] |  |  |  |  | Carrying out actions required to engage in education, work and employment and to conduct economic transactions, including the use of technologies. | ICF browser (adapted) |  |  |
|  |  | D8.a Engaging in informal education [ICF: D810] |  |  |  | Participating in learning activities outside the formal school setting that develop a person mentally, morally, or aesthetically. | Merriam-Webster (adapted) |  | C0681345 |
|  |  | D8.a Engaging in informal education [ICF: D810] | D8.a.1 Engaging in cultural education |  |  | The act of or relating to the ideas, customs, and social behavior of a society. | Google dictionary |  |  |
|  |  | D8.a Engaging in informal education [ICF: D810] | D8.a.2 Engaging in play education |  |  | The act of educating someone (especially children) on activities for enjoyment and recreation rather than a serious or practical purpose. | Google dictionary |  |  |
|  |  | D8.a Engaging in informal education [ICF: D810] | D8.a.3 Studying |  |  | Application of the mental faculties to the acquisition of knowledge. | Merriam-Webster Dictionary |  |  |
|  |  | D8.a Engaging in informal education [ICF: D810] | D8.a.4 Teaching |  |  | To cause or help (someone) to learn about a subject. | Merriam-Webster Dictionary (adapted) |  | C0682015 |
|  |  | D8.b Engaging in formal education [ICF: D820] |  |  |  | Giving systematic instruction, at a school or university. | Oxford dictionary (adapted) |  | C0681344 |
|  |  | D8.b Engaging in formal education [ICF: D820] | D8.b.1 Enrolling in school |  |  | Officially register as a student in a course. | Oxford dictionary | C17118 | C0036375 |
|  |  | D8.b Engaging in formal education [ICF: D820] | D8.b.2 Attending school |  |  | The action or process of teaching a child. | Merriam-Webster Dictionary |  |  |
|  |  | D8.b Engaging in formal education [ICF: D820] | D8.b.3 Studying |  |  | Devote time and attention to gaining knowledge of (an academic subject). | Oxford dictionary |  |  |
|  |  | D8.b Engaging in formal education [ICF: D820] | D8.b.4 Teaching |  |  | Give information about or instruction in (a subject or skill). | Oxford dictionary |  |  |
|  |  | D8.b Engaging in formal education [ICF: D820] | D8.b.5 Quitting school |  |  | Leave school, usually permanently. | Oxford dictionary |  |  |
|  |  | D8.b Engaging in formal education [ICF: D820] | D8.b.6 Engaging in educational parenting |  |  | The process of taking care of children's educational well being until they are old enough to take care of this themselves. | Merriam-Webster (adapted) |  |  |
|  |  | D8.b Engaging in formal education [ICF: D820] | D8.b.6 Engaging in educational parenting | D8.b.6.1 Attending school event |  | To go and be present at a school event. | Merriam-Webster (adapted) |  |  |
|  |  | D8.b Engaging in formal education [ICF: D820] | D8.b.6 Engaging in educational parenting | D8.b.6.1 Attending school event | D8.b.6.1.1 Attending Parent Teacher Organization meeting | To go and be present at a Parent Teacher Organization meeting. | Merriam-Webster (adapted) |  |  |
|  |  | D8.b Engaging in formal education [ICF: D820] | D8.b.6 Engaging in educational parenting | D8.b.6.2 Speaking with school staff |  | Expressing one's thoughts and feelings in spoken language to a school employee. | Google dictionary (adapted) |  |  |
|  |  | D8.b Engaging in formal education [ICF: D820] | D8.b.6 Engaging in educational parenting | D8.b.6.3 Visiting class |  | To go and be present at a class session. | Merriam-Webster (adapted) |  |  |
|  |  | D8.b Engaging in formal education [ICF: D820] | D8.b.6 Engaging in educational parenting | D8.b.6.4 Discussing class selection |  | To talk about academic class selection with another person or group. | Merriam-Webster (adapted) |  |  |
|  |  | D8.c Engaging in behaviors related to acquiring, keeping and terminating a job [ICF: D845] |  |  |  | Engage in activities to search for, secure, maintain, or terminate work that a person does regularly in order to earn money. | Merriam-Webster (adapted) |  |  |
|  |  | D8.c Engaging in behaviors related to acquiring, keeping and terminating a job [ICF: D845] | D8.c.1 Seeking a job [ICF: D8450] |  |  | To search for work that a person does regularly in order to earn money. | Merriam-Webster (adapted) |  |  |
|  |  | D8.c Engaging in behaviors related to acquiring, keeping and terminating a job [ICF: D845] | D8.c.1 Seeking a job [ICF: D8450] | D8.c.1.1 Applying to a job |  | To ask formally for work that a person does regularly in order to earn money. | Merriam-Webster (adapted) |  |  |
|  |  | D8.c Engaging in behaviors related to acquiring, keeping and terminating a job [ICF: D845] | D8.c.2 Maintaining a job [ICF: D8451] | |  | Retaining a paid position of regular employment. | Google dictionary |  |  |
|  |  | D8.c Engaging in behaviors related to acquiring, keeping and terminating a job [ICF: D845] | D8.c.2 Maintaining a job [ICF: D8451] | D8.c.2.1 Being absent |  | "The practice of regularly staying away from work or school without valid reason," | Google dictionary |  |  |
|  |  | D8.c Engaging in behaviors related to acquiring, keeping and terminating a job [ICF: D845] | D8.c.2 Maintaining a job [ICF: D8451] | D8.c.2.2 Departing early |  | To go away from work before the usual or expected time. | Merriam-Webster (adapted) |  |  |
|  |  | D8.c Engaging in behaviors related to acquiring, keeping and terminating a job [ICF: D845] | D8.c.3 Terminating a job [ICF: D8452] | |  | To cause a job to end. | Merriam-Webster Dictionary (adapted) |  |  |
|  |  | D8.c Engaging in behaviors related to acquiring, keeping and terminating a job [ICF: D845] | D8.c.3 Terminating a job [ICF: D8452] | D8.c.3.1 Quitting |  | "The act of giving up (a job or position) in a formal or official way." | Merriam-Webster Dictionary |  |  |
|  |  | D8.c Engaging in behaviors related to acquiring, keeping and terminating a job [ICF: D845] | D8.c.3 Terminating a job [ICF: D8452] | D8.c.3.2 Retiring |  | "The withdrawal from one's position or occupation or from active working life." | Merriam-Webster Dictionary |  |  |
|  |  | D8.d Engaging in basic economic transactions [ICF: D860] |  |  |  | Engaging in actions where economic value is provided by exchange between parties. | Merriam-Webster Dictionary (adapted) |  |  |
|  |  | D8.d Engaging in basic economic transactions [ICF: D860] | D8.d.2 Spending |  |  | The act of keeping the financial records of a business or person. | Merriam-Webster Dictionary |  |  |
|  |  | D8.d Engaging in basic economic transactions [ICF: D860] | D8.d.2 Spending |  |  | The act of using an accepted method of payment to pay for something else. | Merriam-Webster Dictionary |  |  |
|  |  | D8.d Engaging in basic economic transactions [ICF: D860] | D8.d.2 Spending | D8.d.2.1 Renting movies |  | To pay money in return for being able to view movies (that belong to someone else). | Merriam-Webster Dictionary (adapted) |  |  |
|  |  | D8.d Engaging in basic economic transactions [ICF: D860] | D8.d.2 Spending | D8.d.2.2 Gambling |  | The act of playing a game in which you can win or lose money or possessions. | Merriam-Webster Dictionary |  |  |
|  |  | D8.d Engaging in basic economic transactions [ICF: D860] | D8.d.3 Saving |  |  | The process of setting aside a portion of current income for future use, or the flow of resources accumulated in this way over a given period of time. | Encyclopaedia Britannica |  |  |
|  |  | D8.d Engaging in basic economic transactions [ICF: D860] | D8.d.4 Structuring |  |  | The act of arranging transactions according to a plan. | Oxford Dictionaries (adapted) |  |  |
|  |  | D8.d Engaging in basic economic transactions [ICF: D860] | D8.d.4 Structuring | D8.d.4.1 Opening account |  | The act of making an account open (typically on anticipation of financial transactions). | Merriam-Webster (adapted) |  |  |
|  |  | D8.d Engaging in basic economic transactions [ICF: D860] | D8.d.4 Structuring | D8.d.4.2 Closing account |  | The occurence of any account that has been closed out or otherwise terminated. | Google dictionary |  |  |
|  |  | D8.d Engaging in basic economic transactions [ICF: D860] | D8.d.5 Investing |  |  | The act of expending money with the expectation of achieving a profit or material result by putting it into financial schemes, shares, or property, or by using it to develop a commercial venture. | Google dictionary |  |  |
|  |  | D8.d Engaging in basic economic transactions [ICF: D860] | D8.d.6 Selling |  |  | To exchange (something) for money. | Merriam-Webster Dictionary. |  |  |
|  |  | D8.d Engaging in basic economic transactions [ICF: D860] | D8.d.7 Acquiring goods and services [ICF: D620] |  |  | The act of acquiring (something) permanently or temporarily. | Google dictionary |  |  |
|  |  | D8.e Using technology |  |  |  | Engaging in a task using technical processes, methods, or knowledge. | Merriam-Webster (adapted) |  |  |
|  |  | D8.e Using technology | D8.e.1 Using computer devices |  |  | The act of using a computer device (electronic machines such as laptop, smartphone, tablet, etc.). | Merriam-Webster (adapted) |  |  |
|  |  | D8.e Using technology | D8.e.1 Using computer devices | D8.e.1.1 Using the internet |  | Use of global computer network providing a variety of information and communication facilities. | Oxford Dictionary |  |  |

|  | D9. Engaging in behavior related to community, social, and civic life [ICF: D9] |  |  |  |  | Engaging in activities related to a unified body of individuals, including recreation, community affairs, and pro/antisocial behavior. | Merriam-Webster (adapted) |  |  |
| --- | --- | --- | --- | --- | --- | --- | --- | --- | --- |
|  |  | D9.a Engaging in community life [ICF: D910] |  |  |  | Engaging in activities with or related to a group of people who live in the same area (such as a city, town, or neighborhood). | Merriam-Webster (adapted) |  |  |
|  |  | D9.a Engaging in community life [ICF: D910] | D9.a.1 Engaging in behavior related to spirituality |  |  | Engaging in behaviors concerned with spirituality, religion or religious matters | Merriam-Webster |  |  |
|  |  | D9.a Engaging in community life [ICF: D910] | D9.a.1 Engaging in behavior related to spirituality | D9.a.1.1 Praying |  | The act of speaking to God especially in order to give thanks or to ask for something. | Merriam-Webster (adapted) |  |  |
|  |  | D9.a Engaging in community life [ICF: D910] | D9.a.1 Engaging in behavior related to spirituality | D9.a.1.2 Reading spiritual text |  | Engaging in activities that provide refreshment of one's mind or body after work, through amusement or play. | Merriam-Webster (adapted) |  |  |
|  |  | D9.a Engaging in community life [ICF: D910] | D9.a.1 Engaging in behavior related to spirituality | D9.a.1.3 Attending services |  | To go and be present at a meeting for worship. | Merriam-Webster (adapted) |  |  |
|  |  | D9.a Engaging in community life [ICF: D910] | D9.a.2 Joining or maintaining association or group membership [ICF: D9101] |  |  | The act of joining or maintaining membership in an organized groups of people who have the same interest, job, etc. | Merriam-Webster |  |  |
|  |  | D9.b Engaging in recreation and leisure activities [ICF: D920] |  |  |  | Engaging in activities that provide refreshment of one's mind or body after work, through amusement or play. | [TheFreeDictionary.com](http://thefreedictionary.com/) |  |  |
|  |  | D9.b Engaging in recreation and leisure activities [ICF: D920] | D9.b.1 Participating in hobbies/activities [ICF: D9204] |  |  | "a pursuit outside one's regular occupation engaged in especially for relaxation" | Merriam-Webster |  |  |
|  |  | D9.b Engaging in recreation and leisure activities [ICF: D920] | D9.b.1 Participating in hobbies/activities [ICF: D9204] | D9.b.1.1 Engaging in unstructured play |  | Engaging in a game that is not happening according to a plan. | Merriam-Webster (adapted) |  |  |
|  |  | D9.b Engaging in recreation and leisure activities [ICF: D920] | D9.b.1 Participating in hobbies/activities [ICF: D9204] | D9.b.1.2 Playing sports [ICF: D9201] | | The act of engaging in a contest or game in which people do certain physical activities according to a specific set of rules and compete against each other. | Merriam-Webster (adapted) |  |  |
|  |  | D9.b Engaging in recreation and leisure activities [ICF: D920] | D9.b.1 Participating in hobbies/activities [ICF: D9204] | D9.b.1.3 Hunting |  | "To pursue and kill (a wild animal) for sport or food." | Google dictionary |  |  |
|  |  | D9.b Engaging in recreation and leisure activities [ICF: D920] | D9.b.1 Participating in hobbies/activities [ICF: D9204] | D9.b.1.4 Collecting |  | Engaging in the process of bringing or gathering something together. | Google dictionary |  |  |
|  |  | D9.b Engaging in recreation and leisure activities [ICF: D920] | D9.b.1 Participating in hobbies/activities [ICF: D9204] | D9.b.1.5 Building models |  | The act or process of making a usually small copy of something by putting together materials. | Merriam-Webster (adapted) |  |  |
|  |  | D9.b Engaging in recreation and leisure activities [ICF: D920] | D9.b.1 Participating in hobbies/activities [ICF: D9204] | D9.b.1.6 Doing crafts |  | Engaging in activities involving skill in making things by hand. | Google dictionary |  |  |
|  |  | D9.b Engaging in recreation and leisure activities [ICF: D920] | D9.b.1 Participating in hobbies/activities [ICF: D9204] | D9.b.1.7 Participating in performing arts | | Participating in types of art (such as music, dance, or drama) that are performed for an audience. | Merriam-Webster |  |  |
|  |  | D9.b Engaging in recreation and leisure activities [ICF: D920] | D9.b.1 Participating in hobbies/activities [ICF: D9204] | D9.b.1.8 Playing videogame |  | The act of playing an electronic game played by means of images on a video screen and often emphasizing fast action. | Merriam-Webster (adapted) |  |  |
|  |  | D9.b Engaging in recreation and leisure activities [ICF: D920] | D9.b.1 Participating in hobbies/activities [ICF: D9204] | D9.b.1.9 Working with photography equipment |  | To make use of equipment relating to, obtained by, or used in photography. | Merriam-Webster (adapted) |  |  |
|  |  | D9.b Engaging in recreation and leisure activities [ICF: D920] | D9.b.1 Participating in hobbies/activities [ICF: D9204] | D9.b.1.10 Participating in lesson |  | To take part in something that is taught. | Merriam-Webster (adapted) |  |  |
|  |  | D9.b Engaging in recreation and leisure activities [ICF: D920] | D9.b.2 Attending Events |  |  | Go to and be present at a planned occasion or activity (such as a social gathering or sports program). | Merriam-Webster (adapted) |  |  |
|  |  | D9.b Engaging in recreation and leisure activities [ICF: D920] | D9.b.2 Attending Events | D9.b.2.1 Attending Movie |  | Attending to a story or event recorded by a camera as a set of moving images and shown in a theater or on television. | Google dictionary |  |  |
|  |  | D9.b Engaging in recreation and leisure activities [ICF: D920] | D9.b.2 Attending Events | D9.b.2.2 Attending Sports event |  | Attending a planned occasion or activity focused on a sport. | Merriam-Webster (adapted) |  |  |
|  |  | D9.b Engaging in recreation and leisure activities [ICF: D920] | D920.b Attending Events | D9.b.2.3 Attending Performing arts [ICF: D902] |  | Attending a creative activity that is performed in front of an audience, such as drama, music, and dance. | Google dictionary |  |  |
|  |  | D9.c Engaging in behavior related to political life and citizenship [ICF: D950] |  |  |  | Engaging in activities related to be a responsible member of a community and/ or to politcal parties (Public Service Employment Act, adapted) | Public Service Employment Act (adapted) |  |  |
|  |  | D9.c Engaging in behavior related to political life and citizenship [ICF: D950] | D9.c.1 Engaging in civic involvement |  |  | An action designed to identify and address issues of public concern. | American Psychological Association |  |  |
|  |  | D9.c Engaging in behavior related to political life and citizenship [ICF: D950] | D9.c.1 Engaging in civic involvement | D9.c.1.1 Donating |  | "The act of freely offering time, goods, money or expertise for a civic involvement." | Google dictionary |  |  |
|  |  | D9.c Engaging in behavior related to political life and citizenship [ICF: D950] | D9.c.1 Engaging in civic involvement | D9.c.1.1 Donating | D9.c.1.1.1 Volunteering | "To contribute, typically by doing a share of the work." | Google dictionary |  |  |
|  |  | D9.c Engaging in behavior related to political life and citizenship [ICF: D950] | D9.c.1 Engaging in civic involvement | D9.c.1.1 Donating | D9.c.1.1.2 Research participation | The act of participating as an individual that is observed, analyzed, examined, investigated, experimented upon, or/and treated in the course of a particular study. | NCI Thesaurus (adapted) | C41189 | C0681850 |
|  |  | D9.c Engaging in behavior related to political life and citizenship [ICF: D950] | D9.c.1 Engaging in civic involvement | D9.c.1.1 Donating | D9.c.1.1.3 Donating money | "To give money in order to help a cause" | Merriam-Webster |  |  |
|  |  | D9.c Engaging in behavior related to political life and citizenship [ICF: D950] | D9.c.1 Engaging in civic involvement | D9.c.1.1 Donating | D9.c.1.1.4 Donating organs | The act of volunteering to give up and transfer an organ, organ part, or tissue from one body to another, for the purpose of replacing the recipient's damaged or failing organ with a working one from the donor. | NCI Thesaurus (adapted) | C15289 | C0029216 |
|  |  | D9.c Engaging in behavior related to political life and citizenship [ICF: D950] | D9.c.1 Engaging in civic involvement | D9.c.1.1 Donating | D9.c.1.1.5 Donating blood | Volunteering to have blood removed for transplantation or other purpose. | NCI Thesaurus (adapted) | C132448 | C0005795 |
|  |  | D9.c Engaging in behavior related to political life and citizenship [ICF: D950] | D9.c.1 Engaging in civic involvement | D9.c.1.2 Voting |  | "The official choice that someone makes in an election, meeting, etc., by casting a ballot, rasing the hand, speaking their choice aloud, etc." | Merriam-Webster |  |  |
|  |  | D9.c Engaging in behavior related to political life and citizenship [ICF: D950] | D9.c.1 Engaging in civic involvement | D9.c.1.3 Joining or maintaining membership in political organizations |  | "Participation in an organization that, on the basis of one or more shared concerns, attempts to influence public policy in its favour." | Encyclopedia Britannica |  |  |
|  |  | D9.c Engaging in behavior related to political life and citizenship [ICF: D950] | D9.c.2 Supporting a political campaign |  |  | "Offering support for an organized effort which seeks to influence the decision making process within a specific group. Often refers to electoral campaigns, wherein representatives are chosen or referendums are decided." | Google Dictionary |  |  |
|  |  | D9.c Engaging in behavior related to political life and citizenship [ICF: D950] | D9.c.2 Supporting a political campaign | D9.c.2.1 Painting slogans |  | The process or art of using paint to mark a brief attention-getting phrase used in advertising or promotion. | Merriam-Webster (adapted) |  |  |
|  |  | D9.c Engaging in behavior related to political life and citizenship [ICF: D950] | D9.c.2 Supporting a political campaign | D9.c.2.2 Hanging posters |  | To suspend a large printed picture containing a political message. | Oxford Dictionary (adapted) |  |  |
|  |  | D9.c Engaging in behavior related to political life and citizenship [ICF: D950] | D9.c.3 Engaging in political activism |  |  | "The use of vigorous campaigning to bring about political change." | Google Dictionary |  |  |
|  |  | D9.c Engaging in behavior related to political life and citizenship [ICF: D950] | D9.c.3 Engaging in political activism | D9.c.3.1 Engaging in protest |  | Engaging in "a compaint, objection, or display of unwillingess usually to an idea or a course of action." | Merriam-Webster |  |  |
|  |  | D9.c Engaging in behavior related to political life and citizenship [ICF: D950] | D9.c.3 Engaging in political activism | D9.c.3.1 Engaging in protest | D9.c.3.1.1 Engaging in Political contact | Communication with a political actor. | Oxford Dictionary (adapted) |  |  |
|  |  | D9.c Engaging in behavior related to political life and citizenship [ICF: D950] | D9.c.3 Engaging in political activism | D9.c.3.1 Engaging in protest | D9.c.3.1.2 Blocking traffic | "The action or process of obstructing movement, progress, or activity within traffic." | Google Dictionary |  |  |
|  |  | D9.c Engaging in behavior related to political life and citizenship [ICF: D950] | D9.c.3 Engaging in political activism | D9.c.3.1 Engaging in protest | D9.c.3.1.3 Engaging in boycotts | "A punitive ban that forbids relations with certain groups, cooperation with a policy, or the handling of goods." | Google Dictionary |  |  |
|  |  | D9.c Engaging in behavior related to political life and citizenship [ICF: D950] | D9.c.3 Engaging in political activism | D9.c.3.1 Engaging in protest | D9.c.3.1.4 Engaging in strikes | The act of stopping work in order to force an employer to comply with demands. | Merriam-Webster (adapted) |  |  |
|  |  | D9.c Engaging in behavior related to political life and citizenship [ICF: D950] | D9.c.4 Protecting the environment |  |  | Keeping the environment from being harmed. | Merriam-Webster (adapted) |  |  |
|  |  | D9.c Engaging in behavior related to political life and citizenship [ICF: D950] | D9.c.4 Protecting the environment | D9.c.4.1 Conserving energy |  | To avoid wasteful use of power. | Merriam-Webster (adapted) |  |  |
|  |  | D9.d Engaging in prosocial behavior |  |  |  | Engaging in behavior which is positive, helpful, and intended to promote social acceptance and friendship. | Oxford Dictionaries |  |  |
|  |  | D9.d Engaging in prosocial behavior | D9.d.1 Providing emotional support |  |  | Engaging in behavior which provides sympathy and encouragement. | Google Dictionary (adapted) |  |  |
|  |  | D9.d Engaging in prosocial behavior | D9.d.2 Providing informational support |  |  | To give help or assistance through providing information. | Merriam-Webster (adapted) |  |  |
|  |  | D9.d Engaging in prosocial behavior | D9.d.3 Providing tangible/pragmatic support |  |  | To give help or assistance through providing something practical that can be touched or felt. | Merriam-Webster (adapted) |  |  |
|  |  | D9.d Engaging in prosocial behavior | D9.d.3 Providing tangible/pragmatic support | D9.d.3.1 Feeding |  | The act of giving food to someone else, often a child. | Merriam-Webster Dictionary |  |  |
|  |  | D9.d Engaging in prosocial behavior | D9.d.3 Providing tangible/pragmatic support | D9.d.3.1 Feeding | D9.d.3.1.1 Artificial feeding | The act of feeding a baby with food other than mother's milk. | Medical dictionary |  |  |
|  |  | D9.d Engaging in prosocial behavior | D9.d.3 Providing tangible/pragmatic support | D9.d.3.1 Feeding | D9.d.3.1.2 Breastfeeding | The act of nursing (a baby) at the breast; suckling. | Google Dictionary (adapted) |  |  |
|  |  | D9.d Engaging in prosocial behavior | D9.d.3 Providing tangible/pragmatic support | D9.d.3.2 Providing financial support |  | To give help or assistance in the form of money. | Merriam-Webster (adapted) |  |  |
|  |  | D9.e Engaging in antisocial behavior |  |  |  | Behaviors that harm or lack consideration for the well-being of others and/or break laws. | [6] |  |  |
|  |  | D9.e Engaging in antisocial behavior | D9.e.1 Engaging in non-aggressive antisocial behavior |  |  | Non-violent behaviors that harm or lack consideration for the well-being of others. | [7] |  |  |
|  |  | D9.e Engaging in antisocial behavior | D9.e.1 Engaging in non-aggressive antisocial behavior | D9.e.1.1 Not disclosing |  | Not making requested information known. | Merriam-Webster (adapted) |  |  |
|  |  | D9.e Engaging in antisocial behavior | D9.e.1 Engaging in non-aggressive antisocial behavior | D9.e.1.2 Exaggerating |  | To enlarge beyond bounds or the truth. | Merriam-Webster Dictionary |  |  |
|  |  | D9.e Engaging in antisocial behavior | D9.e.1 Engaging in non-aggressive antisocial behavior | D9.e.1.3 Lying |  | Making an untrue statement with intent to deceive or to create a false or misleading impression | Merriam-Webster Dictionary |  |  |
|  |  | D9.e Engaging in antisocial behavior | D9.e.1 Engaging in non-aggressive antisocial behavior | D9.e.1.4 Cheating |  | To practice fraud or trickery. | Merriam-Webster Dictionary |  |  |
|  |  | D9.e Engaging in antisocial behavior | D9.e.1 Engaging in non-aggressive antisocial behavior | D9.e.1.5 Sabotaging |  | Engaging in an act of deliberate subversion. | Merriam-Webster Dictionary |  |  |
|  |  | D9.e Engaging in antisocial behavior | D9.e.1 Engaging in non-aggressive antisocial behavior | D9.e.1.6 Swearing |  | The use of offensive words when speaking. | Merriam-Webster Dictionary |  |  |
|  |  | D9.e Engaging in antisocial behavior | D9.e.1 Engaging in non-aggressive antisocial behavior | D9.e.1.7 Carrying a weapon |  | Holding a weapon, a device which primary purpose is to attack or defend against others. | Merriam-Webster Dictionary |  |  |
|  |  | D9.e Engaging in antisocial behavior | D9.e.1 Engaging in non-aggressive antisocial behavior | D9.e.1.8 Accepting stolen property |  | The act of receiving/taking possession of property that does not belong to the possesor. | Merriam-Webster (adapted) |  |  |
|  |  | D9.e Engaging in antisocial behavior | D9.e.1 Engaging in non-aggressive antisocial behavior | D9.e.1.9 Selling stolen property |  | The act of giving or handing over property that does not belong to the possessor in exchange for money. | Merriam-Webster Dictionary |  |  |
|  |  | D9.e Engaging in antisocial behavior | D9.e.1 Engaging in non-aggressive antisocial behavior | D9.e.1.10 Stealing |  | To take the property of another wrongfully and especially as a habitual or regular practice. | Merriam-Webster Dictionary |  |  |
|  |  | D9.e Engaging in antisocial behavior | D9.e.1 Engaging in non-aggressive antisocial behavior | D9.e.1.11 Drug dealing |  | The act of giving or handing over drugs in exchange for money without a license from the state. | Merriam-Webster Dictionary |  |  |
|  |  | D9.e Engaging in antisocial behavior | D9.e.1 Engaging in non-aggressive antisocial behavior | D9.e.1.12 Breaking curfew |  | The act of being on streets or public areas during hours designated as off-limits by an authority (state or parents). | Merriam-Webster (adapted) |  |  |
|  |  | D9.e Engaging in antisocial behavior | D9.e.1 Engaging in non-aggressive antisocial behavior | D9.e.1.13 Using property of others without consent |  | The act of using someone else´s property without owners permission. | [8] |  |  |
|  |  | D9.e Engaging in antisocial behavior | D9.e.1 Engaging in non-aggressive antisocial behavior | D9.e.1.14 Speeding |  | Operating a motorized vehicle at speeds in excess of the posted limit. | [9] |  |  |
|  |  | D9.e Engaging in antisocial behavior | D9.e.1 Engaging in non-aggressive antisocial behavior | D9.e.1.15 Joining or maintaining membership in criminal enterprise |  | The act of joining or maintaining membership in an organized enterprise carrying out illegal activity. | Merriam-Webster (adapted) |  |  |
|  |  | D9.e Engaging in antisocial behavior | D9.e.1 Engaging in non-aggressive antisocial behavior | D9.e.1.16 Illegal downloading of media |  | The act of downloading media (e.g., books, music, or movies) without the sanction of the copyright holder. | Merriam-Webster (adapted) |  |  |
|  |  | D9.e Engaging in antisocial behavior | D9.e.1 Engaging in non-aggressive antisocial behavior | D9.e.1.17 Disturbing the peace |  | The act of being loud or rowdy in public places | [10] |  |  |
|  |  | D9.e Engaging in antisocial behavior | D9.e.1 Engaging in non-aggressive antisocial behavior | D9.e.1.18 Engaging in truancy |  | The action of staying away from school without good reason; absenteeism. | Google dictionary |  |  |
|  |  | D9.e Engaging in antisocial behavior | D9.e.2 Engaging in aggressive behavior |  |  | Behavior that causes or threatens harm to other people. | [11] |  |  |
|  |  | D9.e Engaging in antisocial behavior | D9.e.2 Engaging in aggressive behavior | D9.e.2.1 Breaking and entering |  | The act of entering a residence or other enclosed property through the slightest amount of force, without authorization. | Legal dictionary |  |  |
|  |  | D9.e Engaging in antisocial behavior | D9.e.2 Engaging in aggressive behavior | D9.e.2.2 Damaging property |  | The act of causing damage to or the destruction of public or private property, caused by a person who is not its owner. | Google dictionary |  |  |
|  |  | D9.e Engaging in antisocial behavior | D9.e.2 Engaging in aggressive behavior | D9.e.2.3 Robbing |  | To take money or property from (a person or a place) illegally and sometimes by using force, violence, or threats. | Merriam-Webster Dictionary |  |  |
|  |  | D9.e Engaging in antisocial behavior | D9.e.2 Engaging in aggressive behavior | D9.e.2.4 Intimidating with weapon |  | The act of using an instrument of combat, designed to injure or kill, in an attempt to persuade anothers actions. | Duhaime's Law Dictionary |  |  |
|  |  | D9.e Engaging in antisocial behavior | D9.e.2 Engaging in aggressive behavior | D9.e.2.5 Verbally abusing |  | The act of using words that attack or injure an individual or words that constitute psychological violence. | Google dictionary |  |  |
|  |  | D9.e Engaging in antisocial behavior | D9.e.2 Engaging in aggressive behavior | D9.e.2.6 Encouraging others to fight |  | Prompting others to take part in a violent struggle involving the exchange of physical blows or the use of weapons. | Oxford Dictionaries (adapted) |  |  |
|  |  | D9.e Engaging in antisocial behavior | D9.e.2 Engaging in aggressive behavior | D9.e.2.7 Stalking |  | The act of criminal activity consisting of the repeated following and harassing of another person. | Legal dictionary |  |  |
|  |  | D9.e Engaging in antisocial behavior | D9.e.2 Engaging in aggressive behavior | D9.e.2.8 Threatening |  | The act of saying that you will harm someone or do something unpleasant or unwanted especially in order to make someone do what you want. | Merriam-Webster Dictionary |  |  |
|  |  | D9.e Engaging in antisocial behavior | D9.e.2 Engaging in aggressive behavior | D9.e.2.9 Fighting physically |  | The act of using physical force to try to hurt someone, to defeat an enemy. | Merriam-Webster Dictionary |  |  |
|  |  | D9.e Engaging in antisocial behavior | D9.e.2 Engaging in aggressive behavior | D9.e.2.10 Engaging in sexual assault |  | The act of illegal sexual contact that usually involves force upon a person without consent or is inflicted upon a person who is incapable of giving consent. | Merriam-Webster Dictionary |  |  |
|  |  | D9.e Engaging in antisocial behavior | D9.e.2 Engaging in aggressive behavior | D9.e.2.11 Engaging in domestic violence |  | The act of an escalating set of violent behaviors in the context of a marital or other intimate relationship. | Sourceid: [12] |  |  |
|  |  | D9.e Engaging in antisocial behavior | D9.e.2 Engaging in aggressive behavior | D9.e.2.12 Using a weapon on a person |  | The act of using something to injure, defeat, or destroy on another. | Merriam-Webster Dictionary |  |  |
|  | D10. Engaging in mood / state changing activities and behavior |  |  |  |  | Engaging in behaviors causing changes in the conscious state of mind or predominant emotion. | Merriam-Webster (adapted) |  |  |
|  |  | D10.a Quitting |  |  |  | Discontinuing mood or state changing substance or activity. | Oxford Dictionaries (adapted) |  |  |
|  |  | D10.b Abstaining |  |  |  | Restraining oneself completely from mood or state changing substance or activity. (NOTE: lapse / relapse should be coded here) | Oxford Dictionaries (adapted) |  |  |
|  |  | D10.c Using Anabolic steroids |  |  |  | The use of synthetic steroid hormones that resemble testosterone in promoting the growth of muscle. | [13] |  |  |
|  |  | D10.d Using Ecstasy |  |  |  | The use of a synthetic amphetamine analog C11H15NO2 used illicitly for its mood-enhancing and hallucinogenic properties — called also MDMA | Merriam-Webster (adapted) |  |  |
|  |  | D10.e Using Methamphetamines and amphetamines |  |  |  | "The use of a synthetic drug of the phenethylamine family that causes the nervous system to become more active resulting in increased energy and excitement." | Merriam-Webster Dictionary |  |  |
|  |  | D10.f Using Cocaine |  |  |  | "The use of a tropane alkaloid that is obtained from the leaves of the coca plant." | [14] |  |  |
|  |  | D10.g Using Tobacco |  |  |  | "The use of the tobacco plant leaf and its products." | Walker, H Kenneth-NCBI |  |  |
|  |  | D10.g Using Tobacco | D10.g.1 Using Oral tobacco |  |  | "The use of tobacco that is placed in the mouth between the cheek and the gum." | Merriam-Webster Dictionary |  |  |
|  |  | D10.g Using Tobacco | D10.g.2 Smoking Cigar(ette) |  |  | "The use of a small roll of paper filled with cut tobacco in order to smoke." | Merriam-Webster Dictionary |  |  |
|  |  | D10.g Using Tobacco | D10.g.3 Smoking Water pipe |  |  | "The use of a device for smoking in which the tobacco smoke passes through water before it is inhaled." | Merriam-Webster Dictionary |  |  |
|  |  | D10.h Using Alcohol |  |  |  | "The use of ethanol, a type of alcohol found in beverages, that acts as a central nervous system depressant." | MedicineNet |  |  |
|  |  | D10.i Using Sedatives |  |  |  | The use of an agent that depresses the central nervous system (CNS) and is used to induce calm and sleep. | NCI Thesaurus (adapted) | C29756 | C0020592 |
|  |  | D10.j Using Opiates |  |  |  | "The use of the coagulated juice of the opium poppy." | [15] |  |  |
|  |  | D10.k Using Inhalants |  |  |  | "The use of substances that produce chemical vapors that can be inhaled to induce a psychoactive, or mind-altering, effect." | National institute on drug abuse |  |  |
|  |  | D10.l Using Hallucinogens |  |  |  | "The use of drugs that cause profound distortions in a person's perceptions of reality, including delusions and false notions." | Merriam-Webster dictionary |  |  |
|  |  | D10.m Using Marijuana |  |  |  | "The act of smoking the dried leaves and flowering tops of the pistillate hemp plant that yield THC." | Merriam-Webster dictionary |  |  |
|  |  | D10.n Engaging in suicidal behavior/ suicide |  |  |  | Suicide is the act of taking one's own life on purpose. Suicidal behavior is any action that could cause a person to die, such as taking a drug overdose or crashing a car on purpose. | [16] |  |  |

**References**

1. Holditch-Davis D, Schwartz T, Black B, Scher M. Correlates of Mother-Premature Infant Interactions. Research in Nursing and health. 2007;30(3):333-46.

2. Conner M, Norman P. Predicting Health Behaviour: Open University Press; 2005.

3. Campbell ME, Troyer L. The Implications of Racial Misclassification by Observers. American Sociological Review. 2007;72(5):750-65.

4. McCullagh M, Lusk SL, Ronis DL. Factors Influencing Use of Hearing Protection Among Farmers: A Test of the Pender Health Promotion Model. Nursing Research. 2002;51(1):33-9.

5. WHO. International Classification of Functioning, Disability and Health: ICF. Geneva. Licence: CC BY-NC-SA 3.0 IGO.: World Health organization, 2001.

6. Berger KS. The developing person through childhood and adolescence: Macmillan; 2003.

7. Hui K-L, Teo HH, Lee S-YT. The value of privacy assurance: An exploratory field experiment. MIS Quarterly. 2007;31(1):19-33.

8. McCarthy B, Casey T. Love, sex, and crime: Adolescent romantic relationships and offending. American Sociological Review. 2008;73(6):944-69.

9. Stead M, Tagg S, MacKintosh AM, Eadie D. Development and evaluation of a mass media Theory of Planned Behavior intervention to reduce speeding. Health Education Research. 2005;20(1):36-50.

10. Bartlett R, Holditch-Davis D, Belyea M, Halpern CT, Beeber L. Risk and Protection in the Development of Problem Behaviors in Adolescents. Research in Nursing and health. 2006;29:607-21.

11. Fraser MW, Nash JK, Galinsky MJ, Darwin KM. The Making Choices Program: Social Problem-Solving Skills for Children. Chapel Hill, NC: University of North Carolina, Work SoS; 200.

12. Giordano PC, Schroeder RD, Cernkovich SA. Emotions and Crime over the Life Course: A Neo-Meadian Perspective on Criminal Continuity and Change. American Journal of Sociology. 2007;112(6):1603-61.

13. Kuhn CM. Anabolic steroids. Recent progress in hormone research. 2002;57:411-34.

14. Multani PK, Saini N, Kaur R, Sharma P. Biomarkers for drugs of abuse and neuropsychiatric disorders: Models and mechanisms.

15. UNODC. Terminology and Information on Drugs. Second edition ed. Vienna: United Nations Office on Drugs and Crime; 2003.

16. Noorani N, Alavi K, Malakooti SK, Salimi S, Jalali A. Mental Health Services Use among People that Attempt Suicide by Taking a Drug Overdose during the Last Year before their Suicide Commission.
