# Supplementary figures and images for: IC-Behavior: An interdisciplinary taxonomy of behaviors
Source: PLoS One. 2021 Sep 17;16(9):e0252003. doi: 10.1371/journal.pone.0252003 (PMC8448352; doi:10.1371/journal.pone.0252003)

**S5 File. Principal Component Analysis of Past Behavior Frameworks**


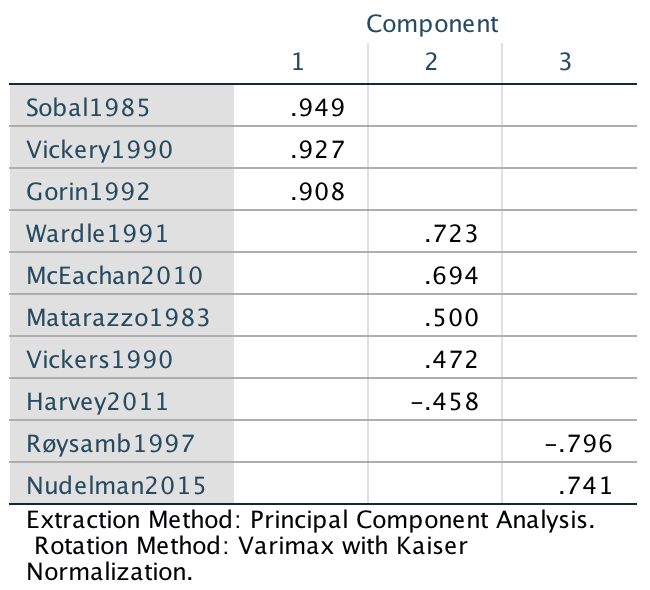

Supplement: S5 File — (DOCX) [file pone.0252003.s005.docx]
